# GHRHR Deficiency Enhances Retinal Ganglion Cell Survival and Visual Functions in Experimental Glaucoma by Inhibiting Ferroptosis

**DOI:** 10.1002/advs.202522929

**Published:** 2026-03-18

**Authors:** Yan Tong, Ming Ho Yam, Jiaxin Zhang, Lin Du, Linbin Zhou, Yolanda Wong Ying Yip, Bo Man Ho, Hemlata Bisnauthsing, Jiahui Li, Ivan Kong, Shushu Xu, Changzhen Fu, Karl K.H. So, Joaquim S.L. Vong, Ling‐Ping Cen, Ming‐Ming Yang, Khazeema Yousaf, Mai Har Sham, Sun On Chan, Poemen P. Chan, Chi Pui Pang, Clement C. Tham, Jing Na He, Jian Li, Wai Kit Chu

**Affiliations:** ^1^ Department of Ophthalmology & Visual Sciences The Chinese University of Hong Kong Hong Kong China; ^2^ Joint Shantou International Eye Center of Shantou University and The Chinese University of Hong Kong Shantou China; ^3^ School of Biomedical Sciences The Chinese University of Hong Kong Hong Kong China; ^4^ Shantou University Medical College Shantou Guangdong China; ^5^ Guangdong Provincial Key Laboratory of Medical Immunology and Molecular Diagnostics, The First Dongguan Affiliated Hospital School of Medical University Dongguan China; ^6^ Dongguan Guangming Eye Hospital Dongguan Guangdong China; ^7^ Department of Ophthalmology Shenzhen People's Hospital (The First Affiliated Hospital, Southern University of Science and Technology; The Second Clinical Medical College, Jinan University) Shenzhen Guangdong China; ^8^ Pakistan Pediatric Association Punjab Pakistan; ^9^ Department of Biotechnology Lahore College for Women University Lahore Pakistan; ^10^ Lam Kin Chung. Jet King‐Shing Ho Glaucoma Treatment and Research Centre The Chinese University of Hong Kong Hong Kong China; ^11^ Department of Ophthalmology, Affiliated Hangzhou First People's Hospital, School of Medicine Westlake University Hangzhou China

**Keywords:** ferroptosis, glaucoma, growth hormone‐releasing hormone receptor, optic neuropathy, single‐cell RNA sequencing

## Abstract

Glaucoma is a leading cause of irreversible blindness worldwide. One hallmark of glaucoma is the degeneration of retinal ganglion cells (RGCs). In this study, a dual role for growth hormone‐releasing hormone receptor (GHRHR) modulation under glaucoma‐relevant conditions and complementary injury paradigms involving the RGCs is identified. Using acute IOP elevation (retinal ischemia‐reperfusion), chronic ocular hypertension (microbead‐induced), and traumatic axonal injury (optic nerve crush) models, we show that GHRHR deficiency preserves RGC survival and uniquely restores visual functions—contrasting with GHRHR activation, which solely promotes cellular survival. Single‐cell transcriptomic analysis uncovers RGC‐specific alterations in genes associated with ferroptosis, lipid metabolism, oxidative stress, and mitochondrial dynamics. At the mechanistic level, GHRHR deficiency prevents the pathological downregulation of key anti‐ferroptotic mediators GPX4 and FTH1 while suppressing pro‐ferroptotic factors ACSL4 caused by glaucomatous neurodegeneration. This multifaceted regulation attenuated iron accumulation, lipid peroxidation, and reactive oxygen species (ROS) accumulation, effects that are diminished by the ferroptosis inducer RSL3. Notably, in mitochondria damaged primary RGCs, pharmacological GHRHR inhibition replicates these benefits, reducing lipid peroxidation and mitochondrial ROS to bolster RGC survival. Collectively, these findings establish GHRHR inhibition as a potent therapeutic strategy for glaucomatous neurodegeneration, synergistically rescuing both structural and functional integrity of the retina.

## Graphical abstract

Glaucoma, a major cause of blindness, involves retinal ganglion cell (RGC) degeneration. This study shows growth hormone‐releasing hormone receptor (GHRHR) deficiency preserves RGC survival and restores vision, unlike activation which only aids survival. Mechanistically, inhibition prevents ferroptosis, reduces oxidative stress, and protects mitochondria. Findings highlight GHRHR inhibition as a promising therapeutic strategy to rescue retinal structure and function in glaucoma.

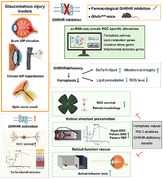

## 1 Introduction

Glaucoma is a heterogeneous group of irreversible vision loss, primarily characterized by the degeneration of retinal ganglion cells (RGCs) and morbid changes in the optic nerve head [1]. It impacts more than 70 million people globally, with approximately 10% bilateral blindness. This number is estimated to exceed 11.1 million by 2040, detrimentally impacting qualities of lives of large number of people worldwide [2]. Elevated intraocular pressure (IOP) is a modifiable risk factor for RGC loss. Current treatments primarily include IOP reduction by topical eyedrops and/or laser trabeculoplasty as the first‐line therapy, minimally invasive glaucoma surgery, and traditional filtration surgeries. Although lowering pathologically high IOP remains the only clinically validated strategy to mitigate the progression of glaucoma, 15%–46% of patients still progress to blindness within 10 to 20 years despite treatment [3]. Furthermore, glaucomatous RGC and axon loss with a constricted visual field can occur even in patients within a physiological range of IOP, suggesting factors beyond the pressure‐mediated damages could also contribute to neurodegeneration [4]. This underscores the complexity of the pathophysiology of glaucoma, and highlights the urgent need to understand the mechanisms underlying RGC loss, as well as the necessity for further research into alternative therapeutic targets.

Growth hormone‐releasing hormone (GHRH) is a hypothalamic peptide that plays a critical role in regulating the synthesis and secretion of growth hormone (GH) through its G protein‐coupled receptor, GHRHR ([5]). GH exerts metabolic effects on target tissues and stimulates the release of insulin‐like growth factor 1 (IGF1) from the liver, which promotes the physiological growth of multiple target tissues, including cartilage and bone [6, 7]. Beyond their well‐established roles in neuroendocrine functions, GHRH and its receptor are involved in many non‐neuroendocrine tissues [8, 9]. Emerging evidence highlights the therapeutic potential of GHRHR antagonists, which are competitive inhibitors of the receptors and demonstrate notable anti‐inflammatory, antioxidant, antitumor, and immunomodulatory properties [8, 10, 11, 12]. GHRH signaling also influences lipid metabolism; notably, GHRHR antagonists have been found to restore the effects of GLP1 on hyperlipidemia by modulating circulating lipid levels [13]. In the visual system, we previously identified detectable levels of GHRH and GHRHR expression in several mouse ocular tissues, suggesting a role of the GHRH signaling pathway in modulating biological functions in eyes at both physiological and pathological ocular conditions [14]. For instance, overexpression of bovine GH reduces the oscillatory potentials of the full‐field flash electroretinogram (ERG) [15], while GHRHR antagonists mitigated ocular inflammation in experimental pterygium and uveitis [16]. Our recent findings showed that manipulating GHRHR with either agonist MR409 or antagonist MIA602 can promote the survival of RGCs in rats challenged by optic nerve crush (ONC). These effects appear to be mediated through the activation of macrophage and suppression of inflammation‐related gene expression, respectively [17]. However, whether RGC preservation translates to functional visual recovery is unknown, and the distinct mechanisms underlying the different regulatory responses of GHRHR agonist and antagonist in the protection of RGCs require further clarification.

Recently, ferroptosis has been identified as a distinct form of cell death characterized by iron‐dependent lipid peroxidation [18]. This process presents an intrinsic vulnerability due to the presence of volatile polyunsaturated lipids on cellular membranes. Accumulation of these membrane lipid peroxides can be lethal to the cells [19]. Ferroptosis is characterized by unique morphological, biochemical, metabolic and genetic features including iron overloading, intracellular antioxidants depletion, lipid peroxidation accumulation and mitochondrial abnormalities [19]. The evidence to date suggests that the development of various subtypes of glaucoma and many other blinding diseases is driven by ferroptosis. For example, pathologically acute high IOP was demonstrated to disturb normal iron homeostasis in RGCs, which is a crucial factor in driving ferroptosis [20]. Recent single‐cell RNA sequencing (scRNA seq) studies have revealed that ferroptosis occurs in various retinal cell types, including photoreceptors, glial cells, and RGCs, following ischemia‐reperfusion (I/R) injury. Importantly, the ferroptosis inhibitor Ferrostatin‐1 was able to reduce RGC death and mitigate the immune response induced by microglia, and protect retinal structure and functions [21]. Additionally, another study reported the involvement of ferroptosis in the pathogenesis of chronic experimental glaucoma and ONC injury, demonstrating inhibition of ferroptosis can effectively alleviate the RGC death [22].

To study the neuroprotective effects generated by the GHRHR signaling, we performed a detailed examination of retinal cells in experimental glaucoma using single‐cell RNA sequencing (scRNA‐seq). Based on the scRNA‐seq results, we further evaluated the critical roles of ferroptosis in experimental glaucoma pathogenesis in vivo, alongside primary RGC cultures in vitro. To anchor glaucoma‐related conclusions to acute paradigms while leveraging chronic ocular hypertension for mechanistic insight, we first employed the retinal I/R mouse model to establish disease relevance and dissect ferroptosis‐related mechanisms under acute IOP elevation, and then used microbead‐induced chronic IOP elevation and ONC to validate the consistency of GHRHR‐mediated neuroprotection across distinct injury contexts. Our findings indicate that the inhibition of GHRHR, either through GHRHR antagonist treatment or *Ghrhr* genetic knockout, significantly enhanced RGC survival and preserved visual functions by suppressing ferroptosis. Conversely, the ferroptosis inducer RSL3 was able to abolish these protective effects conferred by GHRHR inhibition. These findings underscore the potential of targeting GHRHR signaling as a therapeutic strategy to mitigate ferroptosis in glaucoma.

## 2 Results

### 2.1 Increased Expression of GHRHR in the Retina of I/R Mice

To investigate the impact of GHRHR signaling pathway in the pathologically acute high IOP‐induced RGC injury, we first investigated the expression of GHRHR in the retina I/R mouse model. This paradigm replicates critical pathological features, such as oxidative stress and transient hypoperfusion, relevant to glaucomatous neurodegeneration. I/R was established by inducing a temporary interruption of blood flow to the retina, followed by the restoration of circulation (Figure S1a). Immunofluorescence analysis demonstrated that Ghrhr is expressed across retinal layers, including ganglion cell layer (GCL), inner nuclear layer (INL), inner plexiform layer (IPL), and outer plexiform layer (OPL) in the WT‐Sham retinas. Following I/R, Ghrhr expression was significantly upregulated, specifically in the GCL and OPL (Figure S1b,c). These findings suggest that GHRHR signaling is involved in mediating the retinal responses to I/R injury.

### 2.2 GHRHR Deficiency Enhances RGC Survival and Retinal Functions post‐I/R

To investigate the roles of GHRHR in I/R injury, we employed both targeted pharmacological interventions and transgenic mice. Specifically, we employed the GHRHR agonist MR409 and antagonist MIA602 to modulate GHRHR activities. Additionally, we assessed the effects in *Ghrhr^lit/lit^* mice, which carry an amino acid substitution mutation (D60G) in GHRHR [23, 24]. This mutation results in diminished GHRH‐GH‐IGF1 signaling, enabling a focused evaluation of the specific contributions of GHRHR in the context of retinal I/R injury.

**FIGURE 1:**
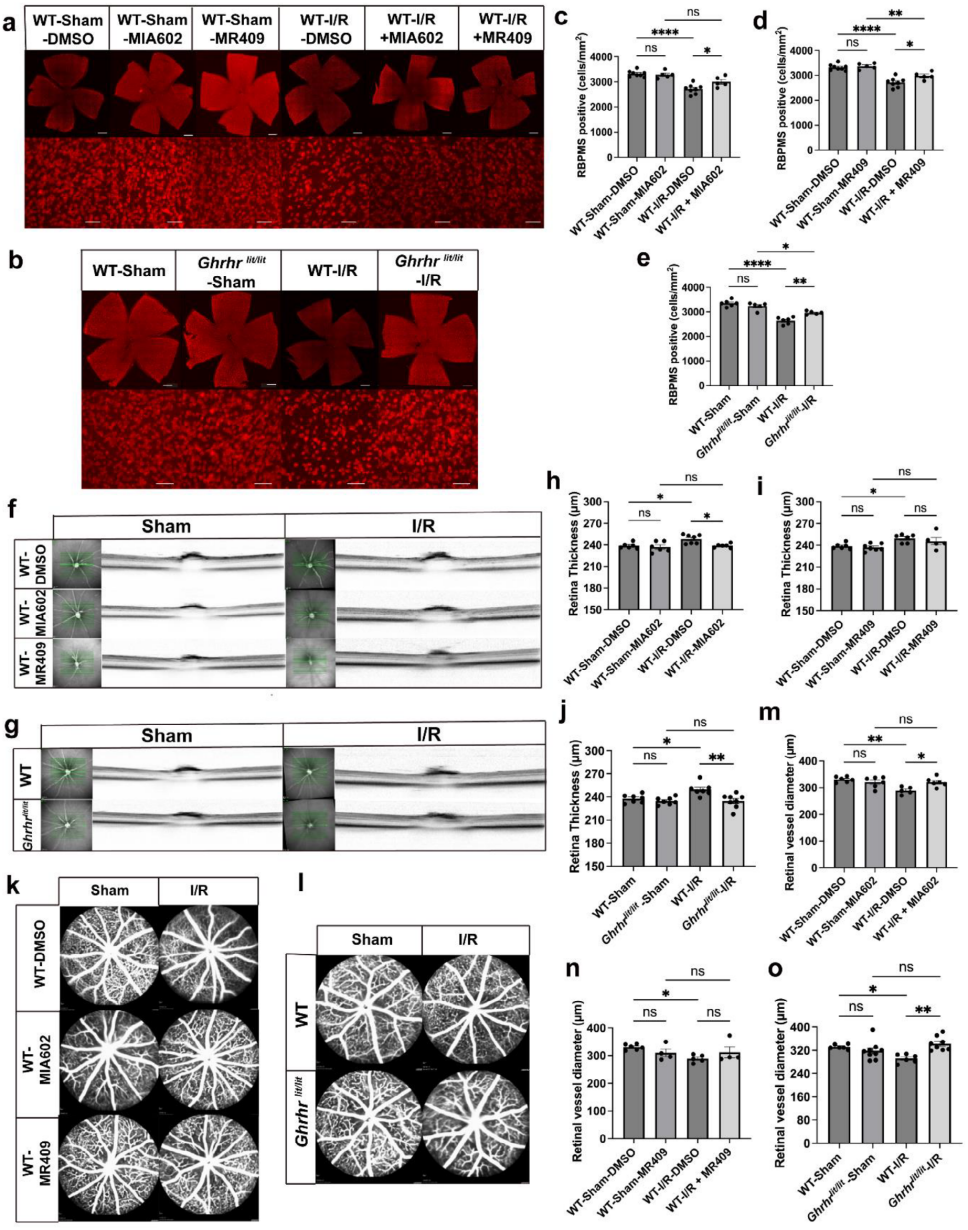
GHRHR inhibition protected against RGC loss, increased retinal thickness, and reduced retinal vessel diameter following I/R injury. (a,b) Representative retinal whole‐mount staining of RBPMS‐positive RGCs in sham challenged mice and mice treated with GHRHR agonist, antagonist, or genetic knockout seven days after I/R (approximately 0.8–1.2 mm from the optic nerve head). (c‐e) Quantification of RBPMS‐positive RGCs across different experimental groups. (f,g) Representative OCT images from retinas five days post‐challenge with or without GHRHR inhibition. (h–j) Quantification of the mean retinal thickness as shown in f and g. (k,l) Representative FFA images from retinas five days post‐challenge with or without GHRHR inhibition. (m–o) Quantification of the mean retinal vessel diameter shown in k and l). N = 4 to 9 mice per group. All results are presented as mean ± SEM; *P*‐values are calculated using one‐way ANOVA with Tukey's correction; significance levels are denoted as *P < 0.05, **P < 0.01, and ****P < 0.0001. ns represents no significance. Scale bars, 500 µm (top row in panel a and b) and 50 µm (bottom row in panel a and b).

To determine whether manipulating GHRHR can protect the retina from I/R induced injuries, we examined retinal morphology and electrophysiology responses after I/R. By immunostaining the retinas for the pan RGC marker, RNA binding protein with multiple slicing (RBPMS), we found that one week following the I/R injury, the number of surviving RGCs was significantly reduced compared to the sham group. The average numbers of surviving RGCs in the sham group and I/R‐induced retinas were 3,320 ± 44 and 2,716 ±67 per mm^2^, respectively (*P* < 0.0001). Notably, subcutaneous administration of either the GHRHR agonist MR409 or the GHRHR antagonist MIA602 independently resulted in a substantial increase in the number of surviving RGCs compared to the I/R group (Figure 1a,c,d). Additionally, H&E staining confirmed that I/R‐induced retinal morphology damage was ameliorated by GHRHR deficiency (Figure S2a). These results were consistent with our previous study in the ONC rat model treated with MIA602 [17]. The *Ghrhr^lit/lit^* ‐I/R group demonstrated a significant increase in RGC survival compared to the wild‐type‐I/R (WT‐I/R) group from 2,643 ± 58 to 2,962 ± 32 per mm^2^ (*P* < 0.005) (Figure 1b,e). Furthermore, we demonstrate that Ghrhr deficiency dramatically decreased TUNEL positive retinal cells one day after I/R injury (Figure S2b,c).

Multimodal in vivo imaging experiments were conducted to assess retinal morphology changes. Fundus imaging showed normal retinal vasculature with well‐perfused arteries and veins; however, post‐ischemic injury, the optic disc appeared pale with blurred boundaries, and several vessels narrowed, accompanied by irregular white patches on the retina in the I/R groups. Notably, the area of these white patches was significantly smaller in GHRHR‐deficient I/R mice compared to the WT‐I/R group (Figure S2d). High resolution spectral domain optical coherence tomography (OCT) revealed significant retinal thickening in the WT‐I/R‐DMSO mice relative to sham‐operated controls (WT‐Sham‐DMSO) 5 days post‐injury (*p* = 0.022). This pathological thickening was markedly suppressed by both MIA602 (*p* = 0.0237) treatment and in *Ghrhr^lit/lit^* mice (*p* = 0.0023), but not by MR409 (Figure 1f–j). Fluorescein angiography (FFA) at the 5‐day endpoint indicated that the mean total retinal vessel diameter was significantly larger in GHRHR‐inhibited mice than in WT mice following I/R injury (*P* < 0.05), with no significant difference observed between the WT‐I/R‐DMSO and WT‐I/R‐MR409 groups (Figure 1k–o).

**FIGURE 2:**
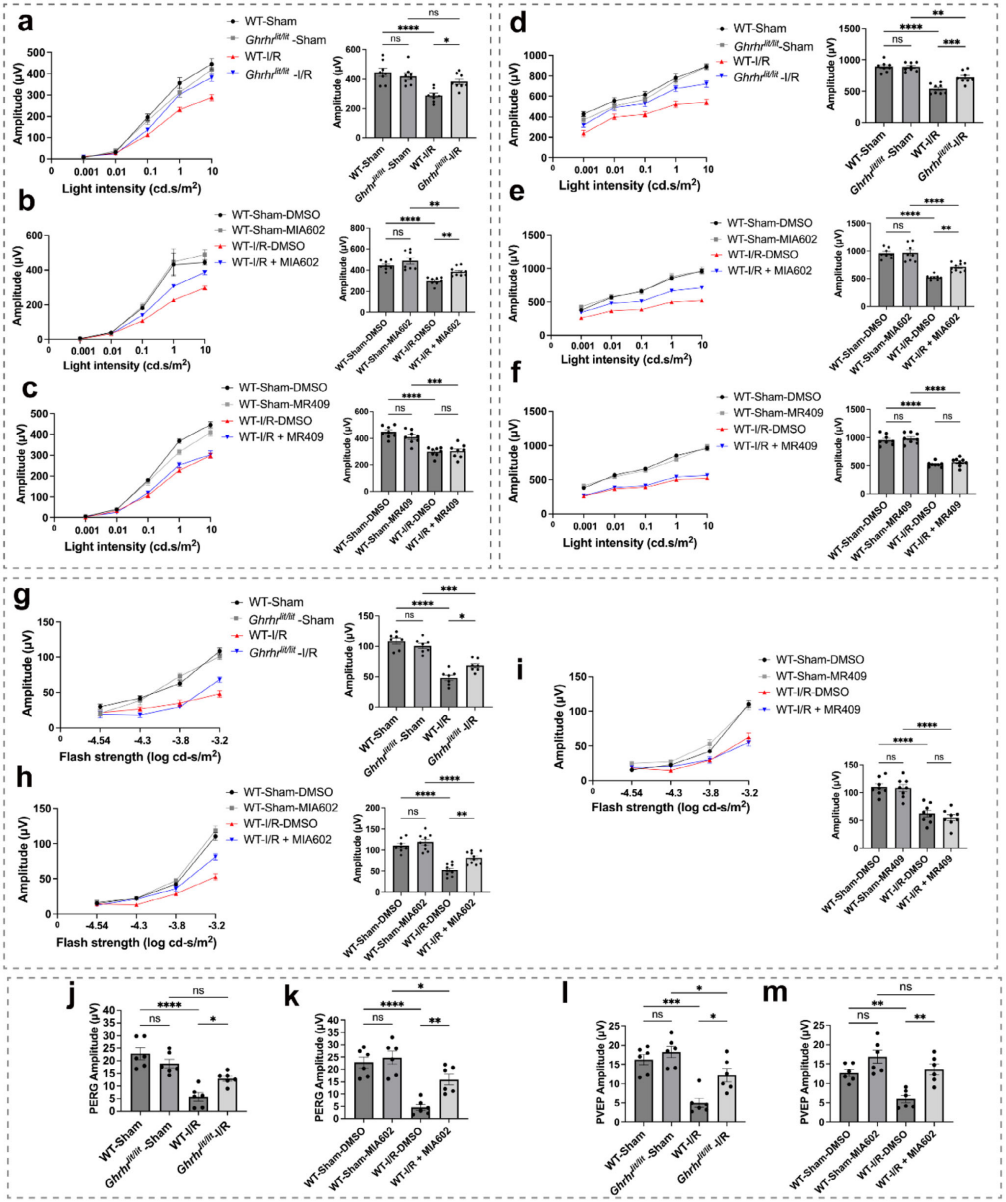
Effect of GHRHR modulation on visual functions in I/R eyes. (a–c) Comparison of amplitudes of scotopic full‐field flash ERG a‐waves recorded from mice in different groups five days post‐I/R challenge. (d–f) Comparison of amplitudes of scotopic full‐field flash ERG b‐waves recorded from mice in different groups five days post I/R challenge. (g–i) Comparison of amplitudes of pSTR waves elicited from mice in different groups five days after I/R. The amplitude of scotopic a‐ and b‐wave were quantified at a light intensity of 10 cd.s/m^2^, while pSTR were quantified at −3.2 log cd‐s/m^2^. (j–m) Analysis of PERG (j and k) and PVEP amplitudes (l and m) in mice with GHRHR inhibition. N = 6 to 8 mice per group; All results are presented as the mean ± SEM; *P*‐values are calculated using one‐way ANOVA with Tukey's correction; significance levels are denoted as *P < 0.05, **P < 0.01, ***P < 0.001, and ****P < 0.0001, ns represents no significance.

Based on our findings that both GHRHR agonist and antagonist could promote RGC survival after retinal I/R, we evaluated global retinal integrity and excluded off‐target effects on non‐RGC retinal circuity by using the dark‐adapted flash electroretinogram (ERG). Amplitudes of both the a‐wave (linked to photoreceptor functions) and b‐wave (associated with rod bipolar cell functions) were reduced significantly in I/R eyes after five days. Consistent to our RGC survival results, GHRHR inhibition (observed in the WT‐I/R‐MIA602 and *Ghrhr^lit/lit^* ‐I/R groups) significantly restored the amplitudes of both a‐ and b‐ waves. However, the treatment of GHRHR agonist MR409 did not improve the a‐ and b‐ wave amplitudes in I/R eyes (Figure 2a–f). Additionally, we assessed the positive scotopic threshold response (pSTR) amplitudes five days post‐I/R, which is an electrical signal originating from the inner retina and is associated with damage of retinal function in glaucoma patients [25]. GHRHR inhibition (by treatment of MIA602 or in *Ghrhr^lit/lit^* mice) mitigated the reduction in pSTR amplitude at day 5 after I/R; however, no improvement was observed in the MR409‐treated I/R group (Figure 2g–i).

Because the full‐field ERG is relatively insensitive to RGC‐specific dysfunction, we next assessed RGC driven visual functions by recording pattern ERG (PERG) and pattern visual evoked potentials (PVEP) to determine whether GHRHR inhibition‐mediated preservation of RGC number translates into functional recovery in experimental glaucoma. Both metrics are more sensitive indicators than pSTR and can accurately reflect RGC functions and the optic pathway in animal models mimicking glaucomatous retinopathy [26]. Representative waveforms for PERG and PVEP in different groups are displayed in Figure S3. Notably, five days after I/R injury, significantly lower amplitudes of the PERG and PVEP (4.571 ±1.225 and 5.988 ± 0.952 µV, respectively) were observed compared to the sham group (22.70 ± 2.254 and 12.74 ± 0.903 µV, respectively) (*P* < 0.0001). Consistent with the pSTR results, GHRHR inhibition (by treatment of MIA602 or in *Ghrhr^lit/lit^* mice) significantly restored the amplitudes of both PERG and PVEP (WT‐IR+MIA602: 15.90 ± 2.184 and 13.62 ± 1.406 µV, respectively; *Ghrhr^lit/lit^*‐I/R: 13.07 ±1.012 and 12.23 ± 1.695 µV, respectively) (*P* < 0.05), suggesting a preservation of RGC function (Figure 2j–m).

In summary, our findings show that while both GHRHR agonist and antagonist can increase the number of surviving RGCs, only the GHRHR antagonist or GHRHR genetic knockout could rescue retinal functions.

### 2.3 Single‐cell RNA Sequencing Revealed Retinal Cell Profiles in WT and *Ghrhr^lit/lit^* Mice With I/R Injuries

To investigate the molecular mechanisms underlying the protective effects of GHRHR inhibition against I/R injury and generate a comprehensive retinal cell atlas, single‐cell RNA‐sequencing (scRNA‐seq) was conducted on mouse retinas from the WT‐I/R and *Ghrhr^lit/lit^* ‐I/R mice. Our analysis revealed 15 transcriptionally distinct molecular clusters, consistently clustering various cell types into distinct regions.

**FIGURE 3:**
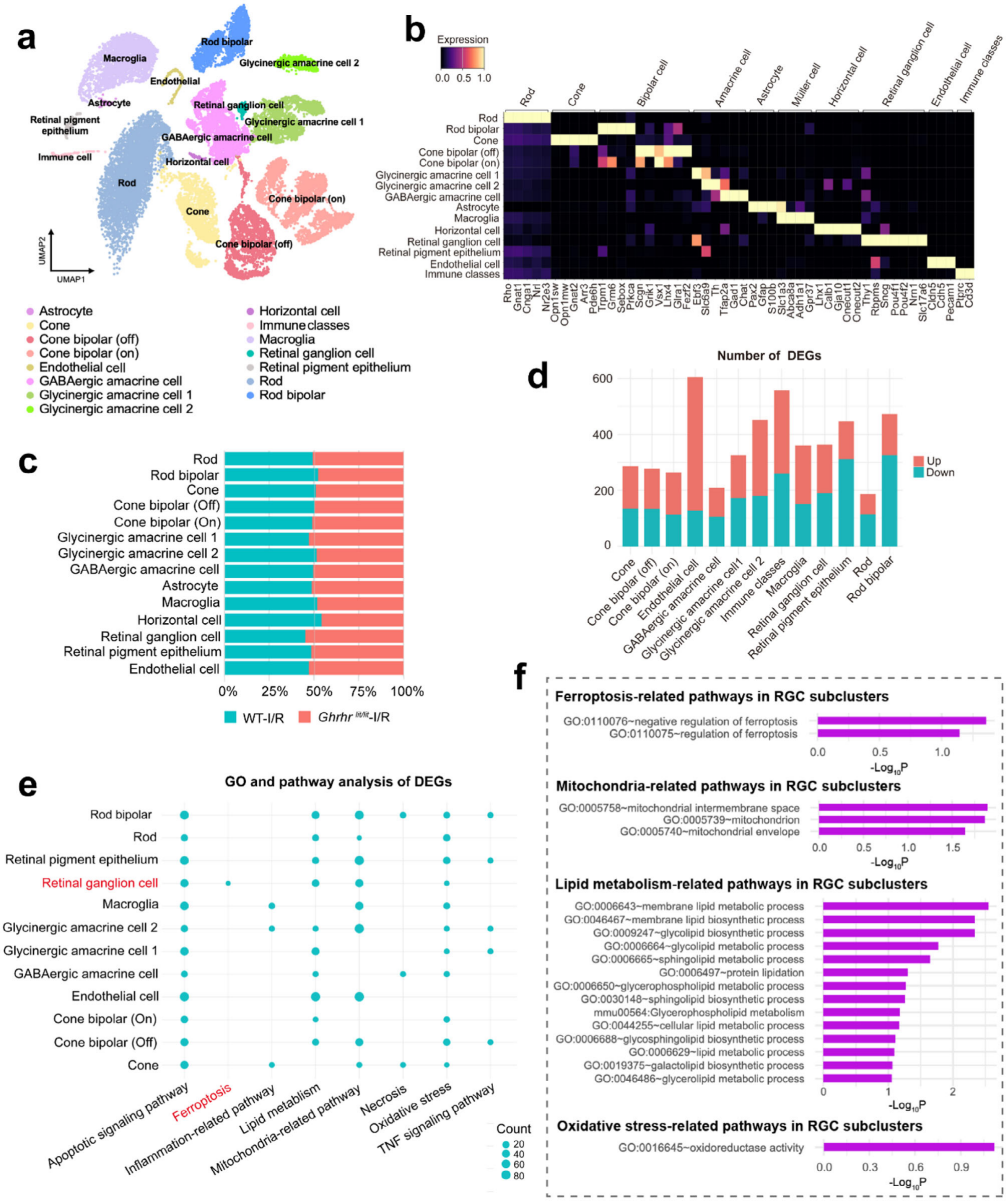
scRNA‐seq revealed altered retinal heterogeneity between WT and *Ghrhr^lit/lit^* mice following I/R injury. (a) Clustering strategy for major retinal cell populations, identifying 15 distinct cell types through the unsupervised clustering combined with the marker‐based clustering analysis. (b) Scaled expression heatmap of canonical markers corresponding to each cluster. (c) Relative cell abundance among each cell cluster between WT‐I/R and *Ghrhr^lit/lit^*‐I/R groups. (d) Number of DEGs that are up‐ or down‐regulated across cell clusters between WT‐I/R and *Ghrhr^lit/lit^*‐I/R groups. (e) Molecular function GO analysis of DEGs between the two groups across various cell clusters. (f) Molecular function GO analysis of DEGs between the two groups across RGC cluster.

We analyzed the distribution of different cell lineages in the retina, encompassing seven neuronal classes: rods, cones, cone bipolar cells, rod bipolar cells, horizontal cells, amacrine cells, and RGCs. We also studied other non‐neuronal populations, including two glial classes (astrocytes and microglia), one immune class and endothelial cells, based on specific markers and the most variable changed genes (Figure 3a,b). The relative abundance of each cell cluster between the WT‐I/R and *Ghrhr^lit/lit^* ‐I/R groups are depicted in Figure 3c and Table S1. We conducted differentially expressed gene (DEG) analysis comparing the two groups to investigate the transcriptional changes caused by the deficiency of GHRHR. The number of up‐ and down‐ regulated DEG is displayed in Figure 3d. Subsequent gene ontology (GO) and pathway analyses revealed the biological implications of the altered genes (Figure S4). Additionally, we showed that these altered genes in various subtypes of retinas between *Ghrhr^lit/lit^*‐I/R and WT‐I/R were mainly enriched in pathways related to cell death regulation (including apoptosis, ferroptosis and necrosis), inflammation (including TNF signaling pathways), mitochondrial function, lipid metabolism and oxidative stress signaling pathways using the bubble grid plot (Figure 3e). Strikingly, ferroptosis emerged as the most prominently enriched pathway in RGCs, but not in other major cell types (Figure 3e). This provides strong genetic evidence that RGCs are the primary responders to GHRHR‐mediated ferroptosis regulation.

**FIGURE 4:**
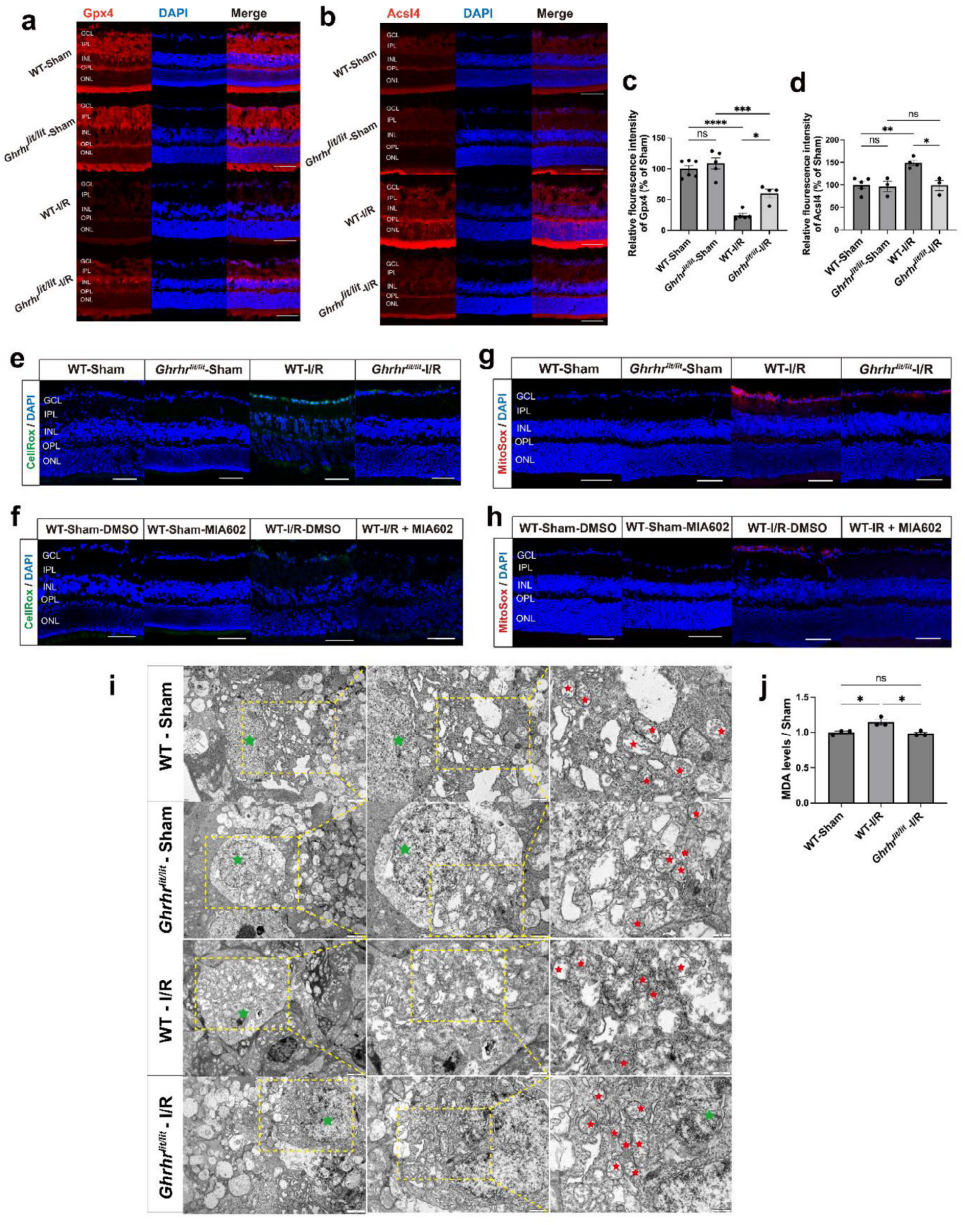
GHRHR inhibition effectively suppressed ferroptosis in mouse retinas following I/R injury. (a,b) Representative retinal immunofluorescence staining for Gpx4 and Acsl4 (red), counterstained with DAPI. (c,d) Relative florescence intensity of Gpx4 and Acsl4 protein expression in retinal sections (N = 3‐6 per group). (e,f) Representative images of CellROX staining in retinas from sham challenge and retinas from one day after I/R injury with GHRHR inhibition. (g,h) Representative images of MitoROX staining in retinas from sham challenge and retinas from one day after I/R with GHRHR inhibition. (i) Representative TEM images of RGCs from WT and *Ghrhr^lit/lit^* mice at 3 days post‐I/R or Sham operation. Images are presented at increasing magnifications from left to right: general ultrastructure (left), cytoplasmic details (middle, region corresponding to the yellow box), and mitochondrial morphology (right). The green stars indicate cell nuclei; red stars mark mitochondria. (j) Measurement of retina MDA levels 3 days after sham and I/R injury in WT and *Ghrhr^lit/lit^* mice (N = 3 per group). *P*‐values are calculated using one‐way ANOVA with Tukey's correction; *, **, ***, and **** represent *p* < 0.05, *p* < 0.01, *p* < 0.001, and *p* < 0.0001, respectively, ns represents no significance. Scale bars: 50 µm (a, b, e–h); 2 µm (i, left), 1 µm (i, middle), 500 nm (i, right).

We identified the RGC cluster based on the expression of Rbpms, which encodes an RNA‐binding protein with multiple splicing, and *Thy 1*, which encodes the cell surface glycoprotein also known as CD90. The DEG analysis of RGCs between *Ghrhr^lit/lit^*‐I/R and WT‐I/R groups revealed over 300 significantly altered genes, visualized in a volcano plot (Figure 4d; Figure S5a). In addition to ferroptosis, GO analysis of these DEGs further revealed several key processes including apoptosis, mitochondrial function, oxidative stress, and lipid metabolism in RGC cluster (Figure 3f). These include genes involved in oxidative stress (*Junb, Pycr1, Paox*), lipid metabolism (*Pigs, Smpd4, Pigf, Prkaa1, Pla2g4e, Pnpla6*), and iron uptake and transport (*Pcca*, *Atp6v1d*, and *Hfe*), which exhibited significantly altered expression in *Ghrhr^lit/lit^*‐I/R group compared to WT‐I/R group (Figure S5b–d). Furthermore, close examination of DEGs revealed that a series of mitochondrial marker genes (such as *Opa1, Abcd3, Ciapin1, Ndufs1, Ndufa9, Ndufa12, Ndufb8, Timmdc1, Pigs, Pcca, Cox20*) also showed significant alterations in the *Ghrhr^lit/lit^*‐I/R group compared to WT‐I/R group (Figure S5e).

Based on these findings, we constructed an integrative transcriptional atlas encompassing multiple retinal neuron cell populations and established a cellular profile to deepen our understanding of the dynamic changes occurring in these cells during GHRHR deficient retinas in response to the I/R challenge. These findings underscore roles of the GHRHR signaling pathway in modulating ferroptosis in the retina following I/R.

### 2.4 Involvement of Ferroptosis in RGC Injury in Mice Following Retinal I/R

To investigate the occurrence of ferroptosis in I/R‐treated retinas, we assessed the protein expression of several key regulators of ferroptosis at various time points post‐I/R. Under un‐challenged conditions, the crucial ferroptosis inhibitor glutathione peroxidase 4 (Gpx4) exhibited the detectable expression across the GCL, INL, and ONL, while the promoter of ferroptosis acyl‐CoA synthetase long‐chain family member 4 (Acsl4) was predominantly localized to the GCL and INL. Following I/R injury, immunofluorescence staining revealed a significant reduction in the levels of Gpx4 at 8 h post‐I/R, with a continued decline observed at 3 days after I/R (Figure S6a,c). We further measured factor ferritin heavy polypeptide 1 (Fth1), another important anti‐ferroptotic factor that serves as a primary intracellular iron storage protein, which showed a similar trend. Western blot analysis confirmed a comparable decrease in their protein levels after I/R (Figure S6f–h). In contrast, levels of Acsl4 increased markedly 1 h after I/R compared to the WT‐Sham group (Figure S6b,d). Additionally, RT‐qPCR results indicated downregulation of anti‐ferroptosis markers *Slc7a11*, *Gpx4*, and *Fth1* at the mRNA level in the I/R induced retinas (Figure S6e).

Extensive evidence has established that elevated iron level, acting as a potent pro‐oxidant, drive ferroptotic cell death through Fenton chemistry [27]. We assessed retinal ferroptosis by measuring the total iron content alongside oxidative stress‐related biochemical markers malondialdehyde (MDA), an end product of lipid peroxidation. Our results demonstrated retinal iron concentrations were markedly elevated one and three days after I/R compared to the WT‐Sham group (Figure S6i). Additionally, a significant increase in MDA levels three days post‐I/R was also detected (Figure S6j).

Taking together, I/R injury induced characteristic morphological and molecular alterations associated with ferroptosis, including the dynamic regulation of Gpx4 and Fth1, upregulation of Acsl4, and increases in both iron contents and lipid peroxidation levels in retina.

### 2.5 GHRHR Inhibition Mitigated I/R‐induced RGC Injury by Suppressing Ferroptosis and Preserving Mitochondrial Functions

We further investigated the role of GHRHR modulation in ferroptosis‐related I/R injury. Immunostaining assays revealed that *Ghrhr^lit/lit^* ‐I/R retinas exhibited increased levels of anti‐ferroptosis marker Gpx4, in the ganglion cell layer, inner nuclear layer, and outer nuclear layer (Figure 4a,c). This upregulation correlated with improved functional outcomes, as evidenced by increased a‐, b‐, and pSTR‐wave amplitudes observed in flash EGR results (Figure 2). We also measured the Fth1 marker which showed a similar trend. These results were validated by western blot analysis (Figure S7a–c). In contrast, the expression of Acsl4 was elevated after I/R challenge and it was significantly reduced in the *Ghrhr^lit/lit^* ‐I/R retina (Figure 4b,d). To determine whether this ferroptosis‐suppressing effect was specific to GHRHR inhibition, we also evaluated the impact of GHRHR agonist treatment. Notably, while the agonist MR409 promoted RGC survival, it failed to mitigate the ferroptotic molecular signature. The immunostaining demonstrated that MR409 treatment did not reverse the I/R‐induced downregulation of Gpx4 and Fth1, nor did it suppress the upregulation of Acsl4. Statistically, the expression levels of these ferroptosis markers in the MR409‐treated group were indistinguishable from those in the I/R group (Figure S7). This dissociation between structural survival and ferroptosis markers in the MR409 group likely explains the lack of functional recovery observed in the ERG recordings.

Given that the suppression of ferroptosis and the associated functional preservation was unique to the GHRHR inhibition strategy, we focused our subsequent analysis exclusively on the GHRHR deficient groups. To further assess oxidative stress, we employed the ROS‐sensitive probe CellROX Green. GHRHR inhibition demonstrated significant antioxidant effects, significantly reducing the accumulation of intracellular ROS, particularly in the RGC layer, in both the WT‐I/R‐MIA602 and *Ghrhr^lit/lit^* ‐I/R groups compared to the WT‐I/R controls (Figure 4e,f).

As the metabolic site of ROS generation in most mammalian cells, mitochondrial are susceptible to lipid peroxidation. Alterations in mitochondrial morphology and functions, such as mitochondrion‐mediated ROS overproduction, impaired ATP synthesis and metabolic reprogramming and contributed to ferroptosis [28]. We assessed mitochondrial ROS production using the MitoSOX Red superoxide as an indicator. Our results demonstrated a significant increase in mitochondrial ROS in the RGC layer one day after I/R. Notably, pharmacological (MIA602) and genetic (*Ghrhr^lit/lit^*) GHRHR inhibition effectively lower mitochondrial ROS levels, indicating improved mitochondrial homeostasis following I/R injury (Figure 4g,h). To further validate the occurrence of ferroptosis and assess the protective effects of GHRHR deficiency at the ultrastructural level, we performed transmission electron microscopy (TEM) on retinal sections (Figure 4i). In both the WT‐Sham and *Ghrhr^lit/lit^* ‐Sham groups, RGCs displayed normal ultrastructural architecture: nuclei appeared oval with evenly distributed chromatin and no signs of pyknosis; the cytoplasm was replete with abundant organelles, and mitochondria exhibited healthy, distinct double membranes with densely packed, well‐organized cristae. No obvious apoptotic bodies or dilated endoplasmic reticulum were observed. In contrast, 3 days post‐I/R injury, RGCs showed nuclear pyknosis and loose cytoplasmic structure. Crucially, mitochondria displayed distinct ferroptotic features [22], including significant shrinkage, increased membrane density, and the rupture or vanishing of mitochondrial cristae. Some mitochondria also exhibited swelling and vacuolization. Remarkably, these ferroptotic ultrastructural changes were significantly attenuated in the *Ghrhr^lit/lit^* ‐I/R group. The RGCs in GHRHR‐deficient retinas largely maintained their structural integrity, presenting regular nuclear morphology and rich cytoplasmic matrices. While slight mitochondrial swelling and minor cristae reduction were noted, the majority of mitochondria retained a preserved shape and membrane structure compared to the WT‐I/R group, confirming that GHRHR inhibition effectively mitigates mitochondrial damage associated with ferroptosis.

Consistent with this, the MDA levels were significantly decreased in retinas isolated from *Ghrhr^lit/lit^* eyes compared to WT controls three days following I/R challenge (Figure 4j). Collectively, these findings suggest that GHRHR inhibition exerts a neuroprotective effect in the context of I/R injury by attenuating ferroptosis, preserving mitochondrial integrity, and reducing oxidative damage.

### 2.6 GHRHR Antagonist Alleviated Rotenone‐induced Mitochondrial Damage and Ferroptosis in Primary RGCs In Vitro

**FIGURE 5:**
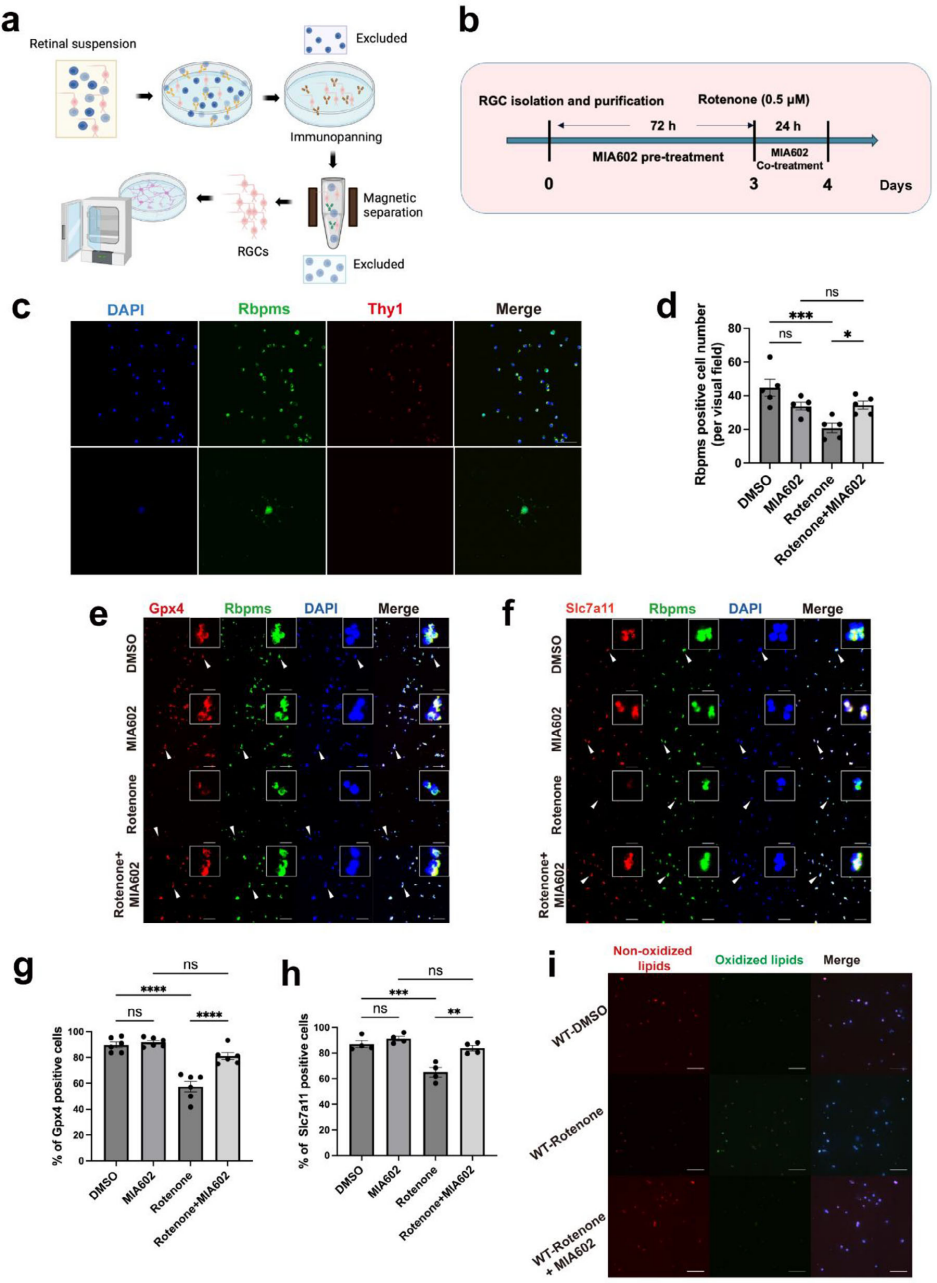
Purified RGCs damaged by mitochondrial poison and rescued by GHRHR antagonist in vitro. (a) Schematic diagram of the isolation and purification of primary RGCs using a two‐step immunopanning method. (b) Experimental timeline of the in vitro RGC treatments. (c) Representative immunofluorescence images of purified RGCs co‐labeled with pan‐RGC markers Rbpms (green) and Thy1 (red). (d) Quantification of Rbpms positive cells per field across treatment groups (N = 5 per group). (e,f) Representative RGC immunofluorescence staining for Gpx4 (red, e) or Slc7a11 (red, f), counterstained with Rbpms (green). (g,h) Relative quantification of the percentage of Gpx4‐positive or Slc7a11‐positive cells (N = 4‐6 per group). (i) Representative images of C11 BODIPY staining in primary RGCs. Data are presented as the mean ± SEM; *P*‐values are calculated using one‐way ANOVA with Tukey's correction; significance levels are denoted as *P < 0.05, **P < 0.01, ***P < 0.001, and ****P < 0.0001. ns represents no significance. Scale bars: 50 µm in (c, e, f, and i). Panel (a) includes graphical elements created with BioRender (www.biorender.com).

While our in vivo findings demonstrate that GHRHR inhibition protects RGCs survival and preserves visual functions, it remains unclear whether this effect is mediated directly through RGC‐intrinsic mechanisms or indirectly via modulation of the retinal microenvironment. We investigated the cell‐autonomous role of GHRHR signaling in regulating ferroptosis in purified primary RGCs. We purified RGCs from the retinas of rats using the two‐step immunopanning protocol (Figure 5a,b). Immunostaining confirmed high purity (>95%) of RGCs based on the co‐expression of pan‐RGC markers Thy1 and Rbpms (Figure 5c). To model mitochondrial dysfunction and ferroptosis, we treated RGCs with rotenone. Rotenone is a potent inhibitor of mitochondrial complex I, leading to disrupted electron transport chain function, increased ROS production, and ultimately inducing mitochondrial dysfunction and cell death [29]. Consistent with our in vivo observations, immunostaining revealed that Rbpms^+^ RGCs were significantly more abundant in rotenone and MIA602 co‐treated cultures compared to the rotenone single treatment group (Figure 5d). To specifically confirm the regulation of ferroptosis markers within RGCs, we further performed co‐immunostaining in primary RGCs. As detailed in Figure 5, a significant reduction in the number of Gpx4*^+^* RGCs in the rotenone‐treated group compared to the dimethyl sulfoxide (DMSO) solvent control treatment (Figure 5e,g). To follow up the canonical Gpx4‐regulated ferroptosis pathway, we assess the level of the upstream marker system xc‐ cystine/glutamate antiporter using Slc7a11, which was also downregulated after rotenone treatment (Figure 5f,h). However, co‐treatment of MIA602 and rotenone rescued the number of Slc7a11*^+^* RGCs, mitigating rotenone‐induced ferroptotic changes. To evaluate lipid peroxidation, an indicator of ferroptosis, we employed the C11 BODIPY fluorescent probe [30]. C11 BODIPY staining demonstrated that rotenone could significantly increase the number of RGCs with oxidized lipid, whereas GHRHR inhibition significantly suppressed this accumulation (Figure 5i). These findings collectively demonstrate that GHRHR inhibition directly protects RGCs by preserving mitochondrial functions, restoring anti‐ferroptotic defences, and reducing lipid peroxidation.

### 2.7 GHRHR Deficiency Enhanced RGC Survival and Retinal Functions in Chronic High IOP Challenged Eyes

**FIGURE 6:**
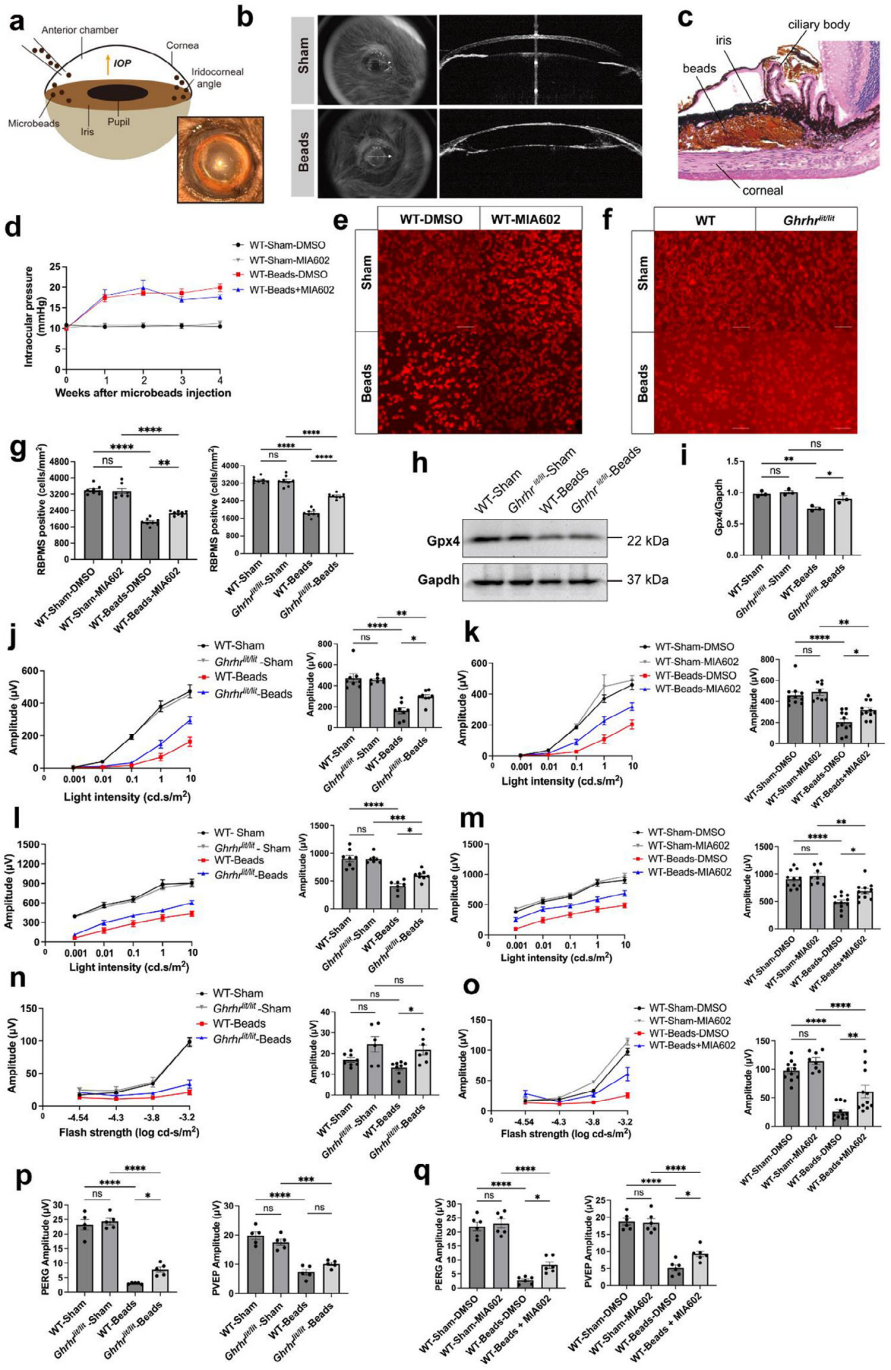
GHRHR inhibition protected RGC loss and retinal functions in a chronic high IOP mouse model. (a) Schematic diagram of chronic high IOP model induction in mouse eyes. (b) Representative ultrasound biomicroscope images of the anterior chamber in sham and beads‐injected eyes. (c) Representative H&E‐stained sections of the anterior chamber in beads‐injected eyes. (d) IOP levels measured over four weeks post‐intracameral injection of magnetic microbeads or PBS (N = 8 per group). (e–g) Representative images (e and f; approximately 0.8–1.2 mm from the optic nerve head) and quantification (g) of RBPMS‐positive RGCs in retinal whole‐mounts from PBS and microbeads injected eyes with or without GHRHR inhibition 30 days post‐beads injection (N = 7‐8 per group). (h,i) Western blot and densitometry analysis of Gpx4 expression in retinas in PBS‐ or beads‐injected eyes 30 days post‐injection (N = 3 per group). (j–o) Amplitudes of scotopic full‐field ERG a‐waves (j, k), b‐waves (l, m), and pSTR (n, o) recorded 30 days post‐beads injection (N = 8‐11 per group). (p,q) PERG and PVEP amplitudes were rescued in *Ghrhr^lit/lit^* eyes (p) and MIA602‐treated mice (q), 30 days post‐injection of microbeads (N = 5‐6 per group). Data are presented as the mean ± SEM; *P*‐values are calculated using one‐way ANOVA with Tukey's correction; significance levels: *P < 0.05, **P < 0.01, ***P < 0.001, and ****P < 0.0001, ns, not significant. Scale bars: 50 µm in (e and f).

To investigate the relevance of glaucoma under chronic IOP elevation, we utilized a well‐established microbead‐induced ocular hypertension model. Magnetic microbeads were injected into the anterior chamber to block the aqueous outflow, as previously reported [22] (Figure 6a), Using this chronic high‐IOP model, we assessed whether GHRHR deficiency preserves RGCs survival, maintains visual functions and suppresses ferroptosis‐associated markers over an extended diseases course. The presence of magnetic microbeads in the anterior chamber was confirmed through H&E staining, slip‐lamp biomicroscope, and ultrasound biomicroscope examinations (Figure 6b,c). In all included eyes, magnetic microbeads remained confined to the anterior chamber angle throughout the observation period. Compared to phosphate‐buffered saline (PBS)‐injected eyes, the injection of magnetic microbeads into the anterior chamber of mice resulted in an increase in IOP from baseline levels of approximately 12 mmHg to 22–28 mmHg three days post‐microbeads injection, with IOP remaining elevated for over four weeks (Figure 6d).

By counting RGCs in the whole‐retina flat mount, bead‐injected eyes exhibited a significant decrease in RGC density four weeks after induction compared to the sham group. And we observed that the loss of RGC can be rescued by GHRHR inhibition (by treatment of MIA602 or in *Ghrhr^lit/lit^* mice) (Figure 6e–g). Furthermore, the ERG showed the reduction of amplitudes of a‐ wave, b‐ wave, and pSTR was rescued by GHRHR inhibition (Figure 6j–o). We also observed similar trends in the results of PERG and PVEP amplitudes, which further reinforced the protective role of GHRHR inhibition in maintaining RGC function under the chronic high IOP challenge (Figure 6p,q).

We then measured the protein expression of the ferroptosis regulator Gpx4 in the retina. Gpx4 expression was significantly reduced in the retina of beads‐injected eyes compared to PBS‐injected eyes four weeks later. GHRHR inhibition restored the decreased Gpx4 expression without affecting elevated microbead‐induced IOP (Figure 6h,i).

### 2.8 GHRHR Inhibition Promotes RGC Survival and Retinal Functions in the Optic Nerve Injured Eyes

**FIGURE 7:**
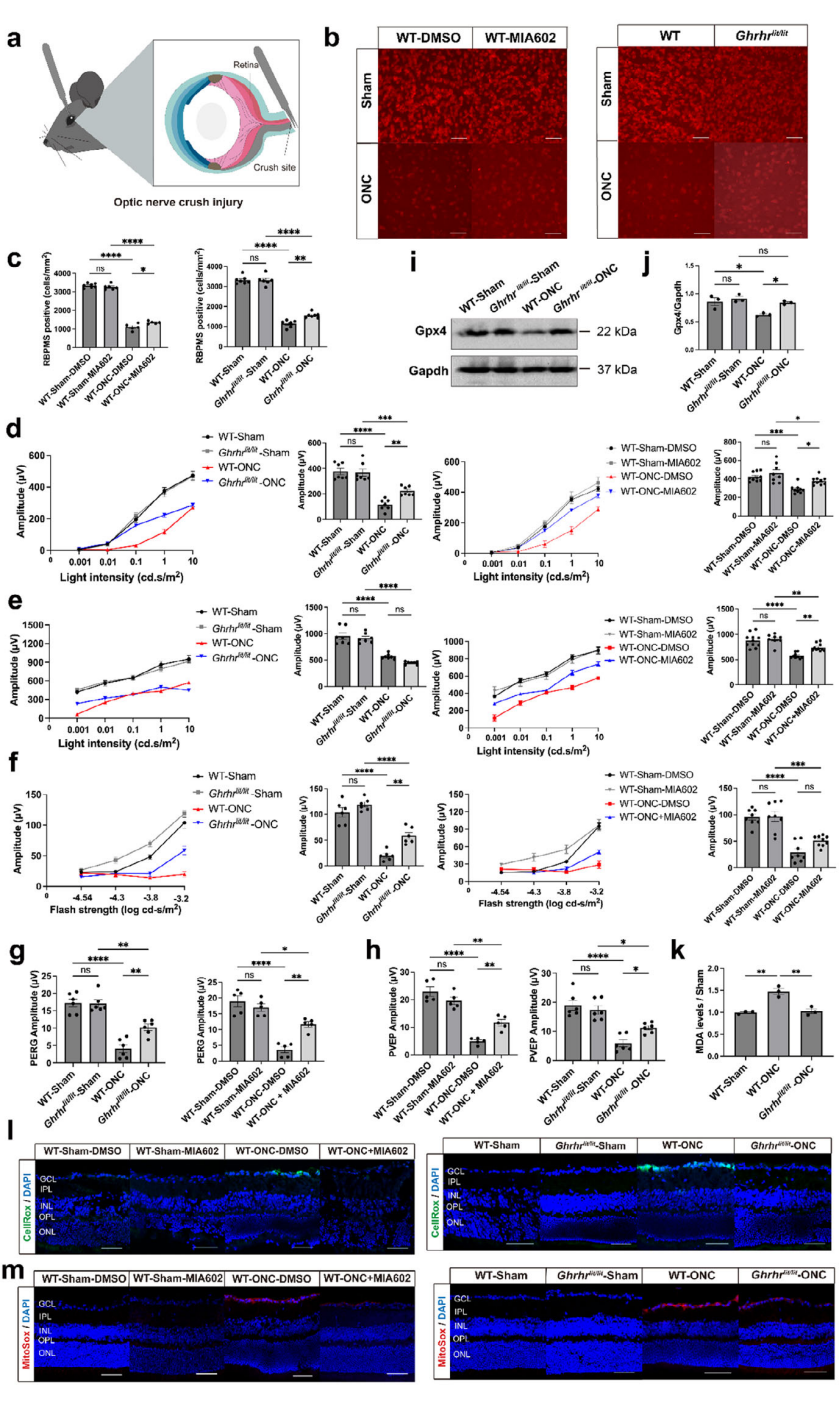
GHRHR deficiency effectively protected RGC loss and inhibited ferroptosis in mouse retinas following optic nerve injury. (a) Schematic diagram of the optic nerve injury generated by ONC damage in mouse eyes. (b,c) Representative immunofluorescence images (b; approximately 0.8–1.2 mm from the optic nerve head) and quantification (c) of RBPMS‐positive cells in whole‐mounted retinas from sham and ONC‐challenged eyes, with or without GHRHR inhibition, 5 days post‐ONC (N = 5‐7 per group). (d–f) Amplitudes of scotopic full‐field ERG a‐waves (d), b‐waves (e), and pSTR (f) recorded 5 days post‐ONC (N = 6‐11 per group). (g,h) PERG (g) and PVEP (h) amplitudes were preserved in *Ghrhr^lit/lit^* retinas and MIA602‐treated mice 5 days post ONC (N = 5‐6 per group). (i,j) Western blot and densitometry analysis of Gpx4 expression in sham and ONC‐injured retinas 3 days post‐injury (N = 3 per group). (k) Measurement of retinal MDA levels in WT and *Ghrhr^lit/li^* mice 3 days post‐ONC (N = 3 per group). (l) Representative images of CellROX staining in retinas from sham and ONC‐challenged *Ghrhr^lit/lit^* or MIA602‐treated mice one day post‐injury. (m) Representative images of MitoROX staining in retinas from sham and ONC‐challenged *Ghrhr^lit/lit^* or MIA602‐treated mice one day post‐injury. Data are presented as the mean ± SEM; *P*‐values are calculated using one‐way ANOVA with Tukey's correction; significance levels: *P < 0.05, **P < 0.01, ***P < 0.001, and ****P < 0.0001, ns, not significant. Scale bars: 50 µm in (b, l‐m).

To further evaluate the neuroprotective role of GHRHR deficiency in acute axonal injury, a hallmark of glaucomatous neurodegeneration, we employed the ONC model. This paradigm induces progressive RGC degeneration and massive cell death, mimicking trauma‐induced glaucoma pathology [31] (Figure 7a). Immunofluorescent staining of the retinal flat mount showed decrease in the densities of RGC 3, 5, and 7 days after ONC compared to the sham group (Figure S8a,b). GHRHR inhibition enhanced RGC survival rates 5 days after ONC in both MIA602 treated and *Ghrhr ^lit/lit^* groups (Figure 7b,c). In addition, the reduced scotopic ERG amplitudes of a‐ wave, b‐ wave, and pSTR were significantly rescued by the inhibition of GHRHR (Figure 7d–f). Furthermore, the diminished amplitudes of both PERG and PVEP were markedly restored with the GHRHR inhibition (Figure 7g,h).

To assess ferroptosis induction in the optic nerve injured eyes, we measured iron and MDA levels in the retinas after ONC. Both iron and MDA levels significantly increased three days after ONC (Figure S8c,d), which was consistent with a previously report [22]. Additionally, Gpx4 expression was markedly downregulated one day after ONC, with further reduction by three days after ONC (Figure S8e,f). *Ghrhr^lit/lit^*‐ONC retinas exhibited significantly higher Gpx4 expression compared to the WT‐ONC group (Figure 7i,j). Moreover, the increase in MDA levels in the ONC retinas was reduced with GHRHR deficiency (Figure 7k). Using the ROS indicator CellRox, we observed increased ROS levels in the ganglion cell layer of mouse retinas one day after ONC compared to the sham control, which was mitigated by the GHRHR inhibition (Figure 7l). Similarly, mitochondrial ROS were significantly elevated in the ganglion cell layer one day after ONC, and the GHRHR inhibition could significantly suppress this in ONC retinas (Figure 7m).

### 2.9 Administration of Ferroptosis Inducer Abolished the Protective Effects Caused by GHRHR Inhibition in Experimental Glaucoma

**FIGURE 8:**
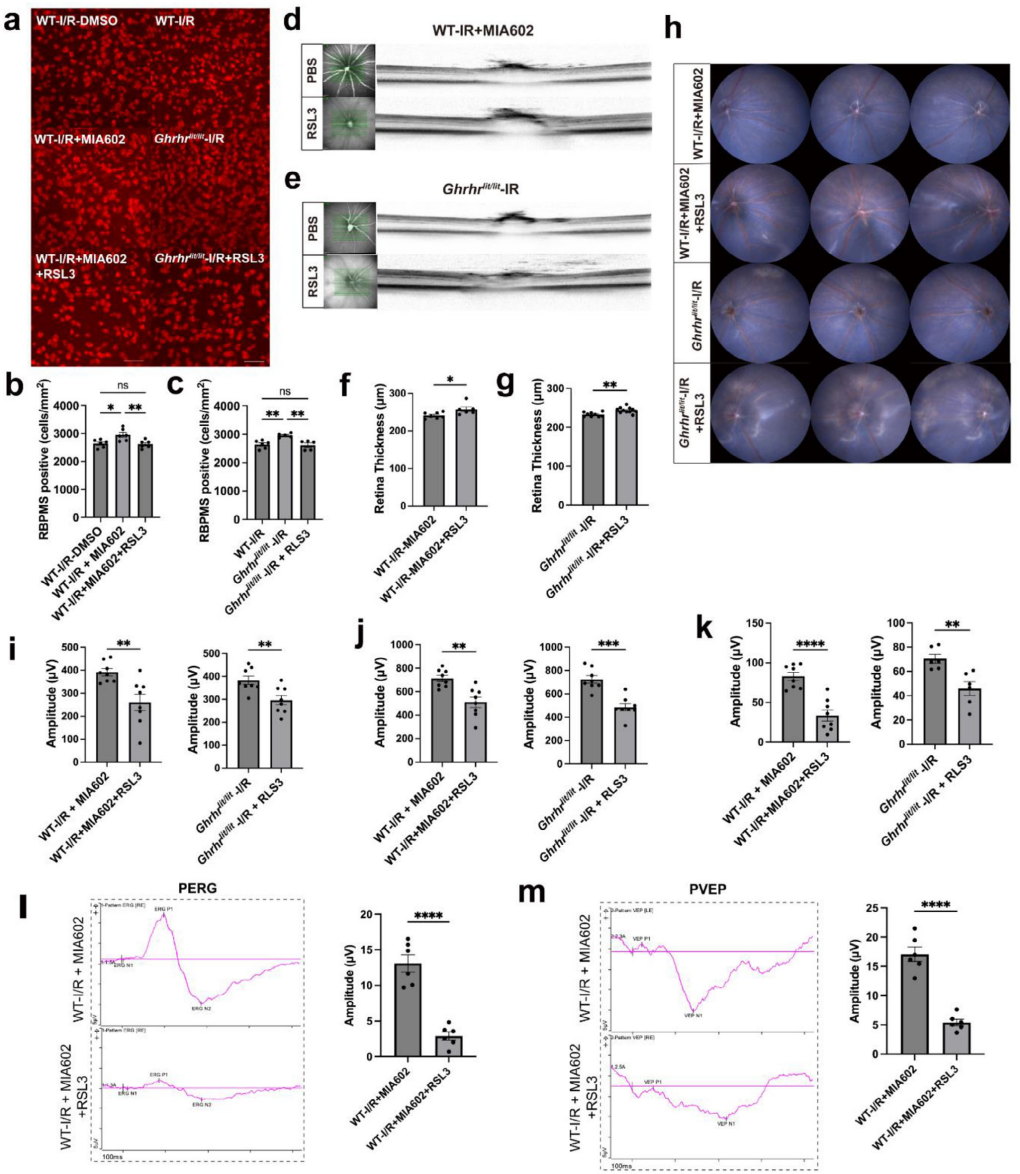
The co‐treatment with ferroptosis inducer RSL3 abolished the protective effects of GHRHR inhibition in the I/R eyes. (a–c) Representative images (a; approximately 0.8–1.2 mm from the optic nerve head) and quantification (b and c) of RBPMS‐positive cells in retinal whole‐mounts from WT‐I/R and GHRHR inhibited eyes plus RSL3 co‐treatment 7 days post‐I/R (N = 5‐6 per group). (d–g) Representative OCT images (d‐e) of retinas and average retinal thickness quantification (f‐g) from GHRHR inhibited eyes plus RSL3 co‐treatment 5 days post‐I/R (N = 6‐11 per group). (h) Fundus photos of *Ghrhr^lit/lit^* mice and MIA602‐treated mice, with or without RSL3 co‐treatment following I/R. (i–k) Amplitudes of scotopic full‐field ERG a‐waves (i), b‐waves (j), and pSTR (k) recorded 5 days post‐I/R (N = 6‐8 per group). (l,m) Representative waveforms and quantification of amplitudes from PERG (l) and PVEP (m) 5 days post‐I/R (N = 6 per group). Data are presented as the mean ± SEM; *P*‐values are calculated using one‐way ANOVA with Tukey's correction (b, c) or unpaired two‐tailed Student's *t*‐tests (f, g, i‐m); significance levels: *P < 0.05, **P < 0.01, ***P < 0.001, and ****P < 0.0001. Scale bars: 50 µm in (a).

To further assess whether the therapeutic effects of GHRHR inhibition were mediated through the regulation of ferroptosis, we intravitreally administered a ferroptosis inducer RSL3, which inactivated the protein Gpx4, alongside MIA602 to observe the treatment outcomes. As shown in Figure 8a–c, while GHRHR deficiency significantly preserved RGC density compared to the vehicle‐treated I/R group, the concurrent application of RSL3 abolished this protective effect. Quantitatively, RGC survival in the co‐treatment group was statistically indistinguishable from that of the WT‐I/R group, indicating that the neuroprotective efficacy of GHRHR inhibition is dependent on suppressing ferroptosis. OCT measurement of retinal structure taken 5 days after I/R challenge suggested that RSL3 significantly abolished the reduction in retinal thickness associated with GHRHR inhibition (Figure 8d–g). Fundus imaging in the retinal I/R mice revealed that the co‐treatment group abolished the repairing of retina damages caused by GHRHR inhibition, characterized by narrower vessels and increased white patches. Similar trends were observed in *Ghrhr^lit/lit^* mice (Figure 8h). Further assessment of retinal functions using flash ERG revealed that co‐treatment with RSL3 abolished the increase in amplitudes of a‐, b‐, and pSTR waves in both WT MIA602‐treated I/R group and the *Ghrhr^lit/lit^* ‐I/R groups (Figure 8i–k). For the RGC functions, RSL3 co‐treatment eliminated the amplitude increases in PERG and PVEP in the WT MIA602‐treated I/R group (Figure 8l,m).

To verify whether the ferroptosis‐dependent mechanism is conserved across different injury paradigms, we extended our investigation to the ONC model. Consistent with our findings in the I/R model, retinal whole‐mount staining demonstrated that treatment with the MIA602 significantly preserved RGC density compared to the DMSO‐treated ONC group. However, this neuroprotective effect was effectively negated by the concurrent administration of the RSL3 (Figure S9a,b). Histological analysis of retinal cross‐sections further confirmed these findings; the number of surviving cells in the GCL was significantly higher in MIA602‐treated retinas, a benefit that was abolished by RSL3 co‐treatment (Figure S9c). Functionally, ERG measurement showed reduced amplitudes of a‐waves, b‐waves, and pSTR in the RSL3 plus MIA602 co‐treatment group five days post‐ONC challenge (Figure S9d–f). These data collectively reinforce that GHRHR inhibition confers neuroprotection against axonal injury via a mechanism dependent on ferroptosis suppression.

### 2.10 GHRHR Inhibition Enhanced Visual Functions in Mice With Experimental Glaucoma

**FIGURE 9:**
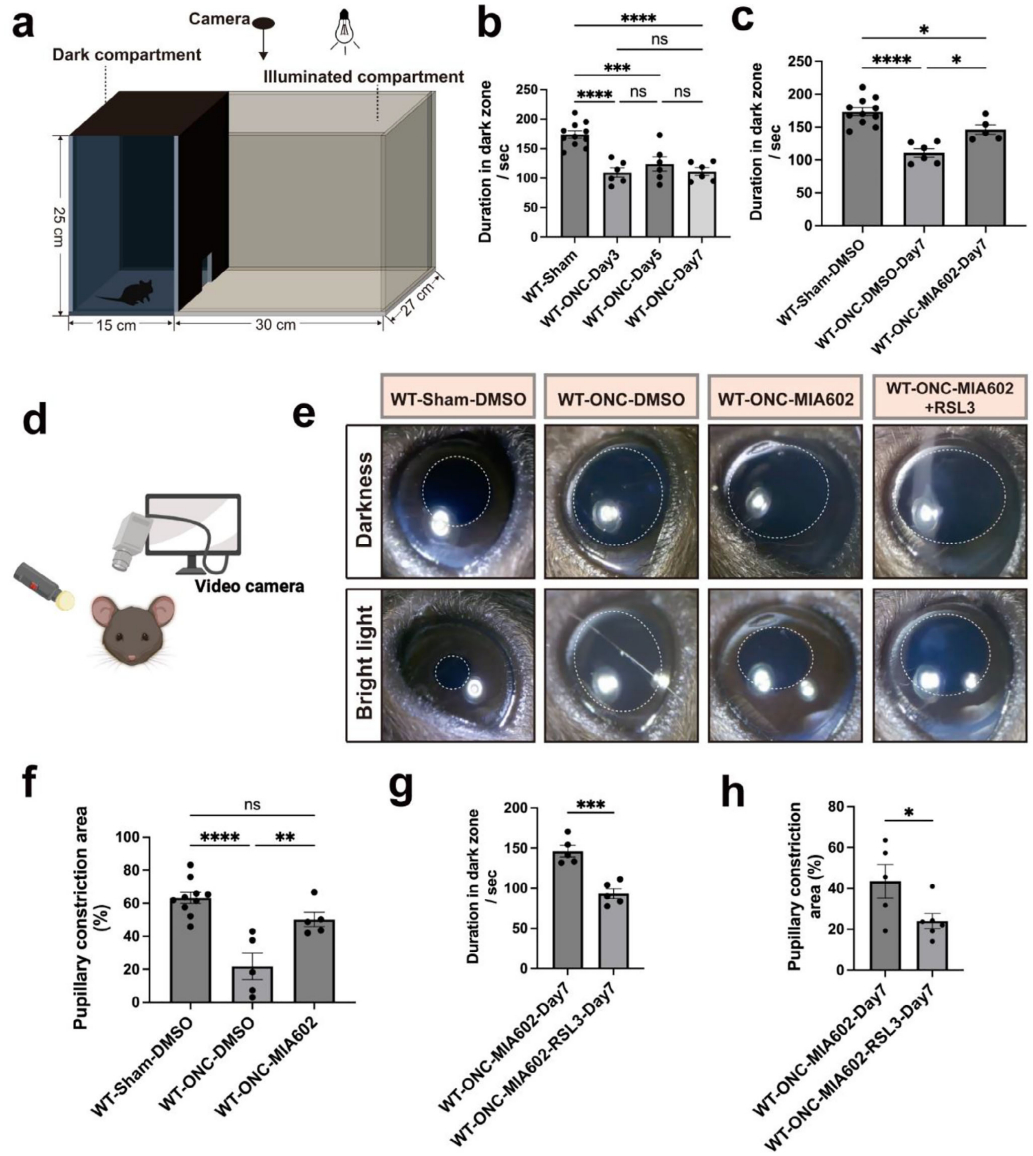
Enhancement of visual functions in GHRHR inhibited mice with experimental glaucoma. (a) The open‐field test box featured a dark zone covering one‐third of the chamber and a larger illuminated zone making up two‐thirds of the chamber. A small opening in the separating wall allowed mice to access both compartments freely. (b) Time spent in the dark zone by sham‐injured mice and mice at different timepoints post‐ONC (N = 6‐11 per group). (c) Time spent in the dark zone by mice treated with or without MIA602 7 days post‐ONC (N = 5‐11 per group). (d) Experimental setup of the pupillary light reflex assessment. Pupil size under light illumination was recorded by a video camera. (e) Representative images of pupil size in dark and light conditions, with white dotted line outlining the pupil areas. (f) Quantification of the pupillary constriction (% area reduction) in mice treated with or without MIA602 3 days post‐ONC (N = 5‐10 per group). (g) Time spent in the dark zone by mice co‐treated with or without RSL3 7 days post‐ONC (N = 5 per group). (h) Quantification of the pupillary constriction (% area reduction) in mice co‐treated with or without RSL3 7 days post‐ONC (N = 5‐6 per group). Data are presented as the mean ± SEM; *P*‐values are calculated using one‐way ANOVA with Tukey's correction (b, c, f) or unpaired two‐tailed Student's *t*‐tests (g, h); significance levels: *P < 0.05, **P < 0.01, ***P < 0.001, and ****P < 0.0001, ns, not significant. Panel (d) includes graphical elements created with BioRender (www.biorender.com).

To evaluate whether visual information transmitted via repaired RGCs can restore visual functions, we conducted two animal behavioral assessments. Given the nocturnal behavior of mice, those with normal vision tended to remain longer in a dark environment, while mice with impaired light perception would spend significantly less time in the dark. We investigated whether GHRHR inhibition could restore this vision mediated behavior in ONC mice using a light/dark box test [32] (Figure 9a). We found that 3–7 days after ONC challenges, mice spent significantly less time in the dark zone (Figure 9b). Following the MIA602 treatment, the time that ONC‐challenged mice spent in the dark zone was significantly increased (Figure 9c). However, this effect was abolished when the ferroptosis inducer RSL3 was co‐administered with MIA602, resulting in reduced time spent in the dark compared to MIA602‐treated ONC mice (Figure 9g).

Subsequently, we performed a pupillary light reflex (PLR) experiment, a visually reflex that operates subcortically [33, 34] (Figure 9d). The PLR signals originate from the neurosensory retina and transmit through the olivary pretectal nuclei, where bilateral input is integrated, and then regulate the pupil sizes of both eyes through the Edinger‐Westphal nucleus of the third cranial nerve [35]. We observed that in sham‐challenged eyes, light stimulation caused a significant decrease in pupil area of over 60%. In contrast, ONC‐treated eyes showed reduced constriction, with pupil area decreased to only 30% following light stimulation. However, MIA602‐treated ONC eyes exhibited a significantly larger pupil constriction area compared to the untreated group, indicating that MIA602 treatment could effectively rescue the pupillary response after ONC (Figure 9e,f). Conversely, MIA602 and RSL3‐cotreated mice demonstrated significantly reduced pupil constriction compared to the MIA602‐treated ONC eyes (Figure 9h).

## 3 Discussion

The prevalence of glaucoma is rising significantly globally, posing a serious threat of irreversible blindness. Currently the pathological mechanisms of glaucoma remain not fully understood, hindering the development of effective treatment strategies [36]. To systematically investigate the roles of GHRHR signaling in glaucoma, we employed a multi‐model approach and disease‐relevant injury paradigms. First, we used *Ghrhr^lit/lit^* mice to investigate the functional consequences of disrupted GHRHR signaling in three experimental glaucoma models. In addition, to dissect its role without confounding developmental or systemic effects, we administered specific GHRHR agonists and antagonists to WT mice to assess the consequences of enhancing or inhibiting GHRHR signaling. Furthermore, to isolate the direct effects of GHRHR on RGCs, we purified primary RGCs and confirmed the cell‐autonomous role of GHRHR in ferroptosis regulation. This integrated experimental strategy provides a robust framework for elucidating GHRHR's multifaceted roles in glaucomatous neurodegeneration.

Various patterns of cell death, including apoptosis, necrosis, autophagy, and pyroptosis, have been implicated in the pathogenesis of glaucoma. However, treatments targeting these processes often only partially mitigate glaucomatous injuries, suggesting that additional cell death pathways may be involved [37, 38, 39]. Previous studies indicated that different modes of cell death play significant roles at distinct time windows. For instance, Rong L. et al. reported that necroptosis, apoptosis, and pyroptosis participated in the process of young and older hepatic I/R injury to various degrees [30], while Cai et al. demonstrated that ferroptosis becomes the predominate driver in the late phase of cardiomyocyte I/R injury [40]. In the present study, we found that GHRHR deficiency significantly ameliorated I/R injury in mice, with the peak protective effects observed during the longer reperfusion period (from 24 h to over 3 days post‐I/R). The delayed protective effect could reflect either the progressive nature of ferroptosis or a broader regulatory role of GHRHR in coordinating multiple cell death pathways. To further delineate the specific modes of cell death at various time windows post injury, future studies should utilize time‐resolved multi‐omics and pathway‐specific inhibitors to dissect the relative contributions of these mechanisms across disease progression. Importantly, our data suggested the need for temporally targeted therapeutic strategies, as GHRHR inhibition may be more effective in later disease stages.

Retinal ferroptosis inhibition has emerged as a promising therapeutic strategy for glaucoma [41], supported by clinical and preclinical evidence of elevated lipid peroxidation [42, 43], and up‐regulated iron‐related proteins in retinas of human glaucoma and experimental monkey glaucoma [44]. However, it remains unclear whether GHRHR regulates ferroptosis in glaucomatous eyes. We demonstrated that GHRHR inhibition alleviated ferroptosis in the experimental glaucomatous retinas by reversing key ferroptosis‐related genes and reducing lipid peroxidation, supporting its roles in both endogenous (enzyme‐regulated) and exogenous (transporter‐dependent) ferroptosis pathways [45, 46]. To further dissect the effects of GHRHR at single‐cell resolution, we employed scRNA‐seq analysis, for the first time, to explore single‐cell transcriptomics in I/R mice with GHRHR genetic knockout. Our DEG analysis revealed a significantly upregulation of *Aifm2*, also known as ferroptosis suppressor protein 1 (*FSP1*), in the *Ghrhr^lit/lit^*‐I/R group. Functionally, *Aifm2* acts as a glutathione‐independent oxidoreductase that regenerates Coenzyme Q10 (CoQ10) into its reduced form, ubiquinol. This reduced CoQ10 serves as a potent radical‐trapping antioxidant within cellular membranes, halting the propagation of lipid peroxidation even when GPX4 activity is compromised [47, 48]. This finding suggests that GHRHR inhibition may engage this compensatory antioxidant mechanisms parallel to the Sl7a11‐Gpx4 pathway, thereby providing a dual‐layered defence against oxidative damage. Notably, our GO analysis aligned with ferroptosis pathophysiology, showing enrichment in oxidative stress, lipid metabolism, and iron homeostasis. These data collectively position GHRHR as a multimodal regulator of RGC survival in experimental glaucoma.

Research into GHRH antagonists has demonstrated their potential in treating conditions such as Alzheimer's disease [49], cancers [50, 51], dyslipidemia [13], and cardiovascular diseases [52], primarily by exerting anti‐oxidation and anti‐inflammatory effects. For instance, long‐term administration of GHRHR antagonist MIA602 and MIA690 in mice could reduce oxidative stress and inflammation while promoting neuroprotection [53]. Given that ferroptosis stems from an imbalance between oxidative damage and antioxidant defence, and often crosstalk with inflammatory signaling, our findings align with this broader protective profile [54, 55]. Specifically, the downregulation of Acsl4 in GHRHR‐deficient retinas may result from the suppression of downstream transcriptional activators, such as Stat3 or Creb, which are key components of inflammatory pathways (for example the Jak‐Stat pathway) known to regulate ferroptosis‐related genes [56]. Additionally, the restoration of Gpx4 and the subsequent reduction in lipid peroxidation may alleviate the stress‐induced upregulation of Acsl4 [57], suggesting a synergistic protection against ferroptosis. Thus, beyond the direct regulation of ferroptosis demonstrated here, the suppression of GHRHR signaling likely modulates broader inflammatory networks (including NF‐kB and cGAS‐STING) during glaucoma progression, offering a multi‐target therapeutic strategy.

Our in vitro findings demonstrated that GHRHR inhibition directly reduces ferroptosis in primary RGCs, suggesting an intrinsic protective mechanism. The observed upregulation of ferroptosis markers (Slc7a11 and Gpx4) and rescue of RGC viability upon GHRHR inhibition provide evidence for this cell‐autonomous effect. However, the possibility of indirect immune‐mediated contributions cannot be excluded. Accumulating evidence indicates that immune‐mediated attacks on the neural retina are key pathological processes underlying glaucoma, with autoreactive T cells playing a central role in the disease progression [58, 59]. Our previous research demonstrated that inhibiting GHRHR in mice reduces the differentiation of T helper 17 cells through the modulation of the JAK‐STAT3 pathway [14]. Additionally, our prior study implicated GHRHR in regulating the activation of microglia, which could subsequently influence RGC survival through neuroinflammatory cascades [17]. While our in vitro system (purified RGC cultures lacking immune cells) supports a direct ferroptosis‐regulatory role, the in vivo milieu likely involves crosstalk between these pathways. Future studies employing conditional knockout models (such as RGC‐specific GHRHR deletion) and immune cells‐RGC co‐culture systems could delineate the relative contribution of cell‐intrinsic versus microenvironmental effects.

Increasing evidence underscores mitochondria as both the initiator and the amplifier of ferroptosis. Cellular ferroptosis induces functional and structural irregularities in mitochondria, which further exacerbate cell susceptibility for ferroptosis, creating a positive feedback loop. This vicious cycle establishes mitochondria as critical hubs for ferroptosis propagation, where initial metabolic insults are exponentially exacerbated through bioenergetic collapse and redox imbalance [60, 61]. To dissect this interplay in RGCs, we pharmacologically disrupted the electron transport chain in primary RGCs by treating them with rotenone and observed that the expression of Gpx4 and its upstream regulator Slc7a11 were upregulated in the GHRHR inhibited cells, accompanied by a significant reduction in oxidized lipid levels and mitochondrial ROS. Previous studies have reported that mitochondrial dysfunction drives ferroptosis through glutamine catabolism and ROS generation [62]. During glutaminolysis, glutamine is converted into the TCA cycle intermediates including α‐ketoglutarate and glutamate within mitochondria, both of which can promote ferroptosis [63, 64]. While the precise molecular mechanisms between GHRHR signaling and mitochondrial metabolic pathways require more in‐depth investigations, our data suggest that GHRHR modulation could decrease ferroptosis sensitivity and disrupt the vicious cycle of mitochondrial damage by mitigating oxidative stress. This protective mechanism may involve both direct regulation of ferroptosis execution elements and indirect effects on mitochondrial ROS generation, positioning GHRHR as a promising target for interrupting this detrimental feedback loop in RGCs.

In our study, pharmacologic modulation of GHRHR increased RGC survival after I/R injury; however, only GHRHR inhibition (MIA602 or *Ghrhr^lit/lit^*) improved retinal functions, whereas agonist did not. Several distinct yet complementary mechanisms may account for this survival‐functional recovery dissociation. First, MR409 may primarily preserve somatic integrity without sufficiently restoring axonal conduction or synaptic output, or it may selectively rescue RGC subtypes that contribute minimally to the pSTR or ERG b‐wave components. Second, although MR409 engages pro‐survival pathways (for example Akt phosphorylation, as previously reported) [65], these signals may be insufficient to counteract the mitochondrial dysfunction and ferroptotic stress characteristic of ischemic injury. In contrast, MIA602 effectively suppresses ferroptosis signatures, a unique property likely essential for re‐establishing the network‐level recovery. Third, the neuroimmune environment may play a pivotal role; prior studies indicate that MR409 can augment microglial activation [17], potentially sustaining a pro‐inflammatory milieu that perturbs Müller cell homeostasis and dampen inner retinal excitability. However, MIA602's anti‐inflammatory effects favor synaptic stabilization and circuit integration. To fully dissect these differential outcomes, future investigations will need to (i) perform RGC subtype–resolved analyses to identify specific populations rescued by each treatment, (ii) rigorously assess axonal and synaptic integrity using anterograde/retrograde tracing and electrophysiological assays, and (iii) clearly define the divergent glial states and inflammation regulatory pathways engaged by MR409 versus MIA602.

While our findings demonstrate GHRHR‐mediated neuroprotection, several limitations warrant consideration. First, although the animal models used here effectively isolate specific injury mechanisms, they represent simplified paradigms that do not fully capture the multifactorial, slow‐progressing pathology of human glaucoma. To bridge this translational gap, future studies will validate the sustained efficacy of GHRHR inhibition in spontaneous glaucoma models (for example DBA/2J mice) and assess clinical relevance in non‐human primates or human‐derived retinal organoids. Second, our study focused exclusively on the receptor (GHRHR) level, without investigating the upstream ligand (GHRH) or directly comparing receptor blockade versus ligand neutralization. Thus, it remains to be determined whether the anti‐ferroptotic effects depend solely on blocking ligand‐receptor interaction or if suppressing GHRHR's constitutive activity also plays a role. Consequently, future investigations will employ genetic approaches, such as GHRH‐knockout models, alongside pharmacological inhibition to dissect the specific contributions of the ligand versus the receptor, thereby fully mapping this signaling landscape.

In summary, our study provides novel and comprehensive evidence that GHRHR plays a pivotal role in the pathogenesis of glaucoma through the mechanism of ferroptosis, and that its inhibition can effectively mitigate glaucomatous damages and preserve visual functions. Furthermore, our in vitro data demonstrate that GHRHR plays a critical role in mitigating mitochondrial ROS and lipid peroxidation, offering neuroprotection for RGCs. These results could be developed into novel therapies by reducing RGC degeneration in glaucoma and other optic neuropathies.

## 4 Methods

### 4.1 Animals

WT mice (C57BL/6J, Jax 000664) and *Ghrhr^lit/lit^* mice (C57BL/6J‐*Ghrhr^lit/lit^* Jax 000533), were purchased from The Jackson Laboratories. *Ghrhr^lit/lit^* mice possess a point mutation in both alleles of the *Ghrhr* gene [14, 23, 66]. Both male and female aged 6 to 8 weeks were used in the study. Sprague‐Dawley rats (both male and female, aged 6 to 8 weeks) were obtained from the Laboratory Animal Service Center of The Chinese University of Hong Kong. All animals were housed under standard conditions, maintained at 22 ± 1°C with 40 ± 10% humidity, and kept on a 12/12‐h dark/light cycle, with free access to food and water and standard laboratory chow ad libitum. All animal experiments were conducted in accordance with guideline of the Association for Research in Vision and Ophthalmology (ARVO) Statement for the use of animals in ophthalmic and vision research. Ethics approval for the study was obtained from the Animal Experimentation Ethics Committee of the Chinese University of Hong Kong (approval number: 22‐269‐MIS and 22‐357‐GRF).

### 4.2 Model of Acute IOP Elevation by Retinal I/R Injury

Retinal I/R was induced by elevating the anterior chamber pressure using a saline‐perfusion system [67]. Briefly, mice were anesthetized using ketamine/xylazine cocktail (100/10 mg/kg, intraperitoneal injection; Ratiopharm, Ulm, Germany). To prevent hypothermia, the anesthetized mice were placed on a heating workbench. A 34‐gauge needle connected to saline bottle was inserted into the anterior chamber through the cornea of the right eye. The IOP was then increased to approximately 80 mmHg for 60 min by elevating the bottle. Retinal ischemia was confirmed by observing the blanching of the retina using an ophthalmoscope. After 60 min, the needle was removed to allow reperfusion of the retina. Contralateral left eyes underwent needle puncture without saline pressurization were served as controls.

### 4.3 Model of Chronic IOP Elevation by Magnetic Microbead Injection

Intracameral injection of magnetic microbeads was performed in the right eye to induce chronic IOP elevation in mice [68, 69]. The anaesthesia procedure was identical to the method described above. A custom‐designed delivery system was utilized, comprising a glass micropipette (BF150‐86‐10, Sutter Instrument, San Francisco, USA) connected to latex tubing and a 1 mL syringe. The cornea was gently punctured near the centre with the micropipette. Approximately 8 × 10^4^ magnetic microbeads (10 µm diameter, concentration 50 mg/mL, BM547, Bangs Laboratories, Indiana, USA) was injected into the eyes, corresponding to a volume of 2 µL. As a control, the contralateral eye received an intravitreal injection of 2 µL PBS. To enhance the retention of the beads, an additional 2 µL of air was injected, creating an air bubble that effectively sealed the injection site and minimized bead loss. Thirty seconds post‐injection, a magnet was employed to position the magnetic beads at the anterior chamber angle. Mice exhibiting signs of inflammation were excluded from the study. IOP was monitored using a rebound tonometer (TonoLab, Icare, Espoo, Finland). IOP readings were taken 2 min after the aesthetic injection and reported as the average of six consecutive measurements. All IOP were measured between 11:00 to 12:00 on the day of the experiment.

### 4.4 Model of RGC Degeneration by ONC

After administering intraperitoneal anesthesia, access to the optic nerve was achieved through an incision in the tissue overlying the superior border of the orbital bone. The superior orbital contents were meticulously dissected, and the rectus muscles were laterally retracted. To improve access to the optic nerve and its surrounding dura mater sheath, the eye was rotated laterally by applying traction to the extraocular muscles. The optic nerve was then exposed within the orbit and subjected to a crush injury using self‐closing forceps for 8 s, positioned approximately 0.5 to 1.0 mm behind the optic disc. The contralateral eyes served as uninjured controls and underwent the same surgical procedure (orbital dissection and nerve exposure) but did not receive the crush injury. Following the procedure, an antibiotic ophthalmic ointment was applied to avoid infection.

### 4.5 Purification and Culture of Primary RGCs

We used the two‐step immunopanning method introduced by Barres et al. to isolate and purify RGCs [27]. Retinal tissues were dissected from the enucleated eyeballs of 6‐week‐old rats and digested enzymatically into single cells using the Papain Dissociation System (Worthington Biochemical Corporation, Cat no. LK003150). We then used the retinal ganglion cell isolation kit (Miltenyi Biotec, Bergisch Gladbach, Germany, Cat no. 130‐096‐209) to purify RGCs. Briefly, single cell suspension was incubated with a biotin‐conjugated antibody, which binds to endothelial cells and microglia, and with CD90.1 MicroBeads labeling RGCs. In the next step, endothelial cells and microglia are magnetically labeled with Anti‐Biotin MACSiBead Particles and depleted by using a MACSiMAG Separator (Miltenyi Biotec, Bergisch Gladbach, Germany, Cat no.130‐042‐102). Subsequently, the cell fraction depleted of endothelial cells and microglia is applied to MS Columns (Miltenyi Biotec, Bergisch Gladbach, Germany, Cat no.130‐042‐201) for positive selection of the CD90.1^+^ RGCs. Upon completion, the column was removed from the separator, and the retained cells were eluted as a RGC fraction.

### 4.6 Drug Treatment

The GHRHR antagonist MIA602 and agonist MR409 were commercially synthesized following established peptide sequences as previously described [50, 70]. For in vivo administration, compounds were first dissolved in 100% DMSO to create stock solutions, then diluted in PBS (DMSO < 0.1%). In experimental models, mice received daily subcutaneous injections of either 20 µg MR409 or 10 µg MIA602 in 100 µL PBS from days 0 to 7 (for I/R and ONC) or day 28 (for beads). Control mice were injected with 0.1 mL PBS containing 0.1% DMSO. For in vitro experiments, primary RGCs were pre‐treated with 1 µm MIA602 for 72 h. Following the pre‐treatment, the cells were co‐cultured with 0.5 µm rotenone for an additional 24 h.

### 4.7 Libraries for Single‐Cell Sequencing

Three days post‐I/R injury, two mouse retinas from each group were harvested to pool as one sample. Two replicates per group were analyzed. After excising the retinas, they were digested enzymatically using Papain and Deoxyribonuclease I (Worthington Biochemical Corporation, Cat no. LK003150). After stopping the digestion, the cell suspension was resuspended in 1 x PBS containing 0.5% bovine serum albumin (BSA). A 10 µL aliquot was stained with Trypan blue for cell counting. The final cell concentration was subsequently adjusted to approximately 1 × 10^6^ cell/mL. Cells were then processed and fixed according to the recommended procedures by 10X Genomics (CG000478). Fixed cells were counted and stored at −80°C and shipped on dry ice to the Single Cell and Spatial Omics Core of School of Biomedical Sciences, The Chinese University of Hong Kong. Fixed RNA Profiling library preparation was performed following the manufacturer's recommendations (CG000527). Libraries were sequenced on a NextSeq 2000 Sequencer (Illumina), achieving a median sequencing depth of 50 000 reads per cell. Sequences were mapped to the mouse genome using the cell ranger multi pipeline in Cell Ranger v.7.0.0.

### 4.8 Single‐Cell RNA Sequencing Analysis

We used 10X Genomics to generate a barcoded scRNA‐seq library of cell suspension samples. The sequencing data was processed using CellRanger software to generate a matrix of unique molecular identifiers that was analyzed using Scanpy.v1.9. Low‐quality cells and genes were filtered for downstream analysis. We conducted stringent filtering (n_genes_by_counts > 700; n_genes_by_counts < 7 000; total_counts < 40 000, pct_counts_mt < 20) to ensure only high‐quality data were retained for downstream analysis. Duplicate cells were also eliminated by Scrublet prediction, with an automatic cutoff of doublet score set at 0.13. 47 043 cells passed this filtering criterion. The feature expression was subsequently normalized with a size factor of 10 000, log‐transformed, and scaled. scRNA‐seq profiles passing quality control were corrected for technical 10 X run batch effects using Harmony software.

We then performed dimensionality reduction and unsupervised clustering using uniform manifold approximation and projection (UMAP). Clusters were annotated using known cell type markers from literature. Selected cell type markers were visualized by heatmap. To identify DEGs between *Ghrhr^lit/lit^*‐I/R and WT‐I/R samples, a pseudo‐bulk differential expression analysis was performed per cell types using the ‘FindMarkers’ function in Seurat v5. In brief, DEGs with absolute log2‐fold change more than 0.5 and a *p*‐value less than 0.05 between cell types of each genotype were assessed.

Functional enrichment analysis was performed using the DAVID web server (http://david.ncifcrf.gov) to identify GO terms (biological processes, cellular components, molecular function), and KEGG pathways significantly altered in the retina of *Ghrhr^lit/lit^*‐I/R mice versus WT‐I/R mice.

### 4.9 Cell Culture and Drug Treatments

Purified RGCs were seeded onto coverslips coated with poly‐D‐lysine and laminin, using pre‐warm RGC growth medium. Cells were then maintained in a humidified incubator at 37°C with an atmosphere of 5% CO_2_ and 95% air. The RGC growth medium formulation is consisted of Neurobasal medium supplemented with the following: BSA (0.1 mg/mL; Sigma), transferrin (0.1 mg/mL; Sigma), progesterone (60 ng/mL; Sigma), putrescine (16 µg/mL; Sigma), selenium (40 ng/mL; Sigma), 3,5,3‐triiodothyronine (T3, 40 ng/mL; Sigma), thyroxine (T4, 40 ng/mL; Sigma), B27 (20 µL/mL; Invitrogen), sodium pyruvate (1 mM; Gibco), glutamine (2 mM; Gibco), N‐acetyl‐L‐cysteine (5 µg/mL; Sigma), insulin (5 µg/mL; Sigma), forskolin (5 µM; Sigma), brain‐derived neurotrophic factor (BDNF, 50 ng/mL; PeproTech, Rocky Hill, NJ), ciliary neurotrophic factor (CNTF, 10 ng/mL; PeproTech), basic fibroblast growth factor (bFGF, 10 ng/mL; PeproTech), and penicillin‐streptomycin (100 U/mL; Gibco).

### 4.10 Live Animal Imaging by Fundus Camera, OCT, and FFA

Following anaesthesia, the pupils of the mice were dilated. A moisturizing gel was applied to protect the ocular surface. Fundus imaging was conducted using a Micron III camera (Micron III, Phoenix Research Laboratories, Pleasanton, CA, USA). During the examination, three photographs were captured, focusing on the central, nasal, and temporal regions of the retina. Retinal‐choroidal thickness of mice was measured and quantified by the OCT imaging system at specific area of the retina which centred on optic disc (Heidelberg Engineering GmbH) as previously described [71]. In addition, fluorescein angiography (FFA) images were analyzed using built‐in software to measure the diameters of major retinal vessels located 5,000 units from the centre of the optic disc. Major retinal vessels were defined as those originating from the optic nerve head and extending to the edges of the fundus images. The total diameters of major retinal vessels for each mouse were averaged based on the number of vessels for statistical analysis.

### 4.11 Dark‐Adapted Full Field Flash Electroretinography (ERG)

Mice were dark‐adapted for at least 12 h before the ERG recording. Pupils were dilated following anesthesia. All procedures were conducted in a dark room illuminated under a dim red light. A gold ring electrode was placed on the cornea, with a reference electrode in the mouth and a ground electrode subcutaneously placed in the hind leg. Visual function was assessed by the Diagnosys Espion system and the ColorDome light‐emitting diode full‐field stimulator (Diagnosys LLC, Lowell, MA, USA). Brief white flashes of varying intensities were presented, with interstimulus intervals controlled by a software. pSTR responses were recorded at flash intensities of ‐4.54, ‐4.3, ‐3.8, and ‐3.2 log cd·s/m^2^, averaging at least 20 responses per recording. Subsequently, a‐waves and b‐waves were measured at intensities of 0.001, 0.01, 0.1, 1, and 10 cd·s/m^2^, with each measurement averaging at least 5 responses.

### 4.12 Pattern ERG (PERG) and Pattern Visual Evoked Potential (PVEP)

Following dark adaptation, mice were anesthetized, and pupil dilation was performed. PERG and PVEP were conducted using the Diagnosys Espion system (Diagnosys LLC, Lowell, MA, USA) with binocular stimulation. Throughout the recording, body temperature was maintained at 37°C. To ensure corneal moisture, a small drop of artificial tears was applied topically as needed. PERG signals were recorded via a subcutaneous needle inserted into the snout, while reference and ground electrodes were positioned medially on the back of the head and at the root of the tail, respectively. For PVEP, the recording electrode was placed in the occipital region, with reference and ground electrodes following the positions as in the PERG setup. PERG data were derived from the average of two consecutive trials, each of which was superimposed 300 times. The grand average PERG waveforms were automatically analyzed using the built‐in software to identify the major positive (P1) and negative waves (N2), and to calculate the sum of their absolute values (peak‐to‐trough) amplitudes. For PVEP, the amplitudes from P1 peak to the N2 trough were measured.

### 4.13 Intravitreal Injection

A 5 µL Hamilton syringe equipped with a 33G needle was employed for intravitreal injections, ensuring careful technique to prevent any damage to the lens of the animals. In C57BL/6J and *Ghrhr^lit/lit^* mice, a 2 µL dose of RSL3 (0.5 mM) (Cat no. SML2234, Sigma) was injected into the vitreous cavity immediately prior to the introduction of retinal I/R and ONC. RSL3 was dissolved in 0.1% DMSO.

### 4.14 Quantification of RGC Survival in Retina

Mice were euthanized, and their eyes were removed and fixed in 4% paraformaldehyde (PFA) for 60 min to prepare retinal flat mounts. The retinas were blocked with 5% bovine serum albumin (BSA) and 0.5% Triton X‐100 in PBS, then incubated with a RBPMS primary antibody (Cat no. GTX118619; GeneTex, Irvine, CA, USA). After three washes in PBS, an Alexa Fluor 594 secondary antibody (anti‐rabbit, Thermo Fisher Scientific, Waltham, MA, USA) was applied. Radial incisions were made to flatten the retinas into a ‘petal’ shape on a glass slide. Images were captured using a fluorescent inverted microscope (Eclipse Ti2, Nikon, Tokyo, Japan) at two fields per petal, specifically 0.8–1.2 mm and 1.8–2.2 mm from the optic nerve head. Surviving RGCs were quantified with ImageJ software (National Institute of Health, USA).

### 4.15 Histological Staining

Hematoxylin and eosin (H&E) staining was performed following standard protocols. Briefly, the eyeballs of mice were fixed in 4% PFA overnight, followed by dehydration through a graded series of ethanol, cleared in xylene, and embedded in paraffin. The paraffin blocks were sectioned into 5 µm slices using a pathological slicer (Leica RM2016, Germany). Sections were rinsed with water after a 5‐min staining period with hematoxylin and eosin solutions. Retinal sections were then examined under a light microscope (DMRB, Leica Microsystems) connected to a Spot digital camera (Diagnostic Instrument).

For IF staining, retinal sections were washed with PBS buffer three times and were blocked with a solution of 2% BSA and 0.1% Triton X‐100 for 1 h at room temperature. The sections were incubated overnight at 4°C with the appropriate primary antibodies followed by incubation with specific secondary antibodies (1:1000) and DAPI for nuclear staining. Images were acquired using the Nikon A1MP confocal microscope using the NIS‐Elements software (Nikon, Japan). Fluorescence intensities were quantified using ImageJ. A list of the primary antibodies utilized in this study is provided in Table S2.

### 4.16 C11 BODIPY Staining

Primary RGCs were rinsed twice with Hank's balanced salt solution (HBSS) before staining. C11 BODIPY 581/591 dye (Cat No. 95978, Cell Signaling Technology, Danvers, MA, USA) was diluted in HBSS to a final concentration of 2 µM and incubated with cells for 30 min at 37°C in dark. After incubation, cells were washed three times with HBSS to remove unbound dye. A Nikon A1MP confocal microscope was employed to visualize and quantify the oxidized and reduced forms of the dye, providing insights into oxidative stress levels within the cells. Detection of the oxidized and non‐oxidized signals was achieved using 581/591 nm (Texas Red filter) and 488/510 nm (FITC filter) wavelengths, respectively.

### 4.17 Assessment of Iron and Malondialdehyde (MDA) Levels

Retinal levels of MDA and iron in homogenized retinal tissues were quantified using an MDA assay kit (Cat No. ab233471, Abcam, Cambridge, UK) and a tissue iron assay kit (Cat No. MAK025, Sigma‐Aldrich, St. Louis, MO, USA). MDA concentrations were measured by assessing the optical density (OD) at 695 nm, while iron levels were determined at 593 nm using a microplate reader (PowerWave XS Microplate Reader, Biotek, VT, USA). Both MDA and iron levels were normalized to the total protein content, which was measured using a BCA assay (Bio‐Rad, USA).

### 4.18 Terminal Deoxynucleotidyl Transferase‐Mediated dUTP Nick‐End Labeling (TUNEL) Assay

TUNEL assays were performed on retinal sections using an In Situ Cell Death Detection Kit, TMR red (Roche, #12156792910). Briefly, retinal sections were prepared 24 h post‐I/R injury. After washing three times with PBS for 10 min each, sections were permeabilized in PBS containing 0.5% Triton X‐100 for 5 min at room temperature. They were then washed twice in PBS for 10 min before incubation with fresh TUNEL detection solution in the dark at 37°C for 60 min. Sections were washed three times with PBS for 10 min and incubated with DAPI for 20 min at room temperature. TUNEL‐stained sections were examined using a confocal laser scanning microscope (A1MP, Nikon, Japan).

### 4.19 Quantitative Real‐Time Polymerase Chain Reaction (qRT‐PCR)

After euthanizing the mice, total RNA was promptly extracted from retina tissues using TRIzol reagent (Thermo Fisher, Cat no. 15596026, Waltham, MA, USA) and dissolved in RNase‐free diethy1 pyrocarbonate (DEPC)‐treated water. RNA of each sample was pretreated with RNase‐free DNase I (Qiagen, Cat no. 79256) and converted to complementary DNA (cDNA) using SuperScript III reverse transcriptase (Thermo Fisher, Cat no. 18080044, Waltham, MA, USA) according to the manufacturer's instructions in an iCycler PCR instrument (Bio‐Rad). The cDNA products of each sample were subsequently proceeded to measurement of gene expression by SYBR Green PCR Kit (Roche, Cat no.04887352001) in a LightCycler 480 II real time‐PCR instrument (Roche Applied Science). The relative gene expression levels were calculated by the 2^−△△Ct^ method and all expression levels were normalized to the endogenous reference gene *β‐actin* and compared to the control group. The specific primer sequences used for qRT‐PCR are detailed in Table S3.

### 4.20 Western Blotting Analysis

Retinal tissues were washed with ice‐cold PBS and lysed in radioimmunoprecipitation assay (RIPA) buffer containing protease inhibitors cocktail (Sigma‐Aldrich, Cat no. 4693124001) and phosphatase inhibitor (Sigma‐Aldrich, Cat no. 4906837001). The cell lysates were centrifuged at 4°C for 15 min and the supernatants were collected. Protein concentration was assessed using a BCA protein assay kit (ThermoFisher, Cat no. 23225). 15–20 µg protein was mixed with SDS‐PAGE sample loading buffer and boiled at 100°C for 5 min. The denatured proteins were separated on 12.5% sodium dodecyl sulfate‐polyacrylamide gels (SDS‐PAGE) using an acrylamide starter kit (Bio‐Rad, #1610183) and subsequently transferred to polyvinylidene difluoride (PVDF) membranes (Merck Millipore, Darmstadt, Germany). Following a blocking step with 5% skim milk for 60 min, the membranes were incubated overnight at 4°C with the primary antibody GPX4 (1:1000, Cat no. ab125066, Abcam), FTH1 (1:1000, Cat no.4393S, Cell Signaling Technology, Danvers, MA, USA), GAPDH (1:5000, Cat no.3683, Cell Signaling Technology, Danvers, MA, USA). After washing with PBST buffer thrice, the membranes were incubated with anti‐mouse IgG or anti‐rabbit IgG (Cell Signaling Technology) HRP‐conjugated secondary antibodies for 2 h at room temperature. Western blots were developed using Super Signal West Pico PLUS Chemiluminescent Substrate (ThermoFisher, #34580) in an enhanced chemiluminescence detection system (Thermo Fisher, MA, USA), and images were analyzed with ImageJ software.

### 4.21 Light/Dark Box Test and Pupillary Light Reflex [72, 73]

The Light/Dark box test was conducted following a published protocol with a cage measuring 45 × 25 × 27 cm, divided into a white open field (30 × 25 × 27 cm, illuminated at 300 lux) and a dark zone (15 × 25 × 27 cm) with no light, separated by a door (5 × 5 cm) [64]. Mice were dark‐adapted for 2 h and placed in the dark chamber for 2 min to acclimate. The door was then opened, allowing free exploration between chambers for 5 min. Time spent in both the dark and illuminated areas was recorded on a video. All experiments were performed between 18:00 and 21:00.

For the pupillary light reflex assessment, mice were dark‐adapted for 30 min before anaesthesia. A halogen light source (100 W, 12 V) from a surgical microscope (Topcon, Japan) provided the stimulus, focused through an 8x objective and aligned perpendicular to the pupil's centre following a published protocol [65]. The light stimulus lasted 20 s, followed by a 60‐s recovery period in darkness to return the pupil diameter to baseline. Pupil responses were recorded using the WinFast PVR photography system, and the area of constricted pupils was analyzed with ImageJ software.

### 4.22 Total and Mitochondrial Reactive Oxygen Species (ROS) Detection

Total and mitochondria‐derived ROS in the retina were measured using CellRox Green (Cat No. C10444, Thermo Fisher Scientific, Waltham, MA, USA) and Mitosox Red (Cat No. M36008, Thermo Fisher Scientific, Waltham, MA, USA). A total of 2 µL of CellRox and 2 µL of Mitosox, both at a concentration of 250 µM, were intravitreally injected into mice 2 h prior to euthanasia. Eyes were collected and fixed in 4% PFA on ice for 1 h while being shielded from light. Eyecups were meticulously dissected and cryosectioned into 14 µm slices on glass slides, then washed with PBS and mounted with DAPI. Images were acquired using the Nikon A1MP confocal microscope.

### 4.23 Transmission Electron Microscopy (TEM)

Retinal tissues were freshly dissected and immediately fixed in 2.5% glutaraldehyde in 0.1 M phosphate buffer (pH 7.4) for 2 h at 4°C, followed by post‐fixation in 1% osmium tetroxide for 1 h. After dehydration through a graded series of ethanol and acetone, the samples were embedded in epoxy resin. Ultrathin sections (70–90 nm) were cut using an ultramicrotome, stained with uranyl acetate and lead citrate, and visualized using a transmission electron microscope (HT7700; Hitachi, Tokyo, Japan). Images were captured at randomly selected fields in the ganglion cell layer (GCL) to assess mitochondrial morphology.

### 4.24 Statistical Analysis

Statistical analyses and visualizations were performed using GraphPad Prism 10 software (GraphPad Software, San Diego, CA). The normality of data distribution was assessed by the *Shapiro‐Wilk* test. No data transformations were applied, and no outliers were excluded from the analyses unless otherwise specified in the figure legends. For comparisons between two groups, normally distributed data were analyzed by unpaired two‐tailed *Student's t*‐tests, while non‐normally distributed data were analyzed using the *Mann‐Whitney U* test. For comparisons involving three or more groups, one‐way analysis of variance (ANOVA) with Tukey's post hoc correction was applied to normally distributed data; non‐normal distributed data were analyzed using the *Kruskal‐Wallis* test with Dunn's post hoc correction (≥3 groups). Continuous data are presented as mean ± standard error of the mean (SEM), with individual data points shown where appropriate. The sample size (n), corresponding to the number of independent biological replicates is noted for each experiment in the figure legends. *P*‐values less than 0.05 were considered statistically significant (*p < 0.05; **p < 0.01; ***p < 0.001; ****p < 0.0001).

## Author Contributions

Y.T., J.N.H., J.L., and W.K.C. conceptualized the study, drafted the initial manuscript, and created figures. M.H.Y., J.Z., L.D., L.Z., Y.W.Y.Y., B.M.H., H.B., J.L., I.K., C.Z.F., S.X., K.K.H.S., and J.S.L.V. performed the experiments and analyzed data. All authors edited and approved the manuscript.

## Funding

This work was supported in part by the Lam Kin Chung. Jet King‐Shing Ho Glaucoma Treatment and Research Centre.

## Conflicts of Interest

The authors declare no conflicts of interest.

## Ethics

Ethics approval for the study was obtained from the Animal Experimentation Ethics Committee of the Chinese University of Hong Kong (approval number: 22‐269‐MIS and 22‐357‐GRF).

## Supporting information




**Supporting File**: advs74883‐sup‐0001‐SuppMat.docx.

## Data Availability

The data that support the findings of this study are available from the corresponding author upon reasonable request.
