# Supplementary material for: GHRHR Deficiency Enhances Retinal Ganglion Cell Survival and Visual Functions in Experimental Glaucoma by Inhibiting Ferroptosis
Source: Adv Sci (Weinh). 2026 Mar 18:e22929. Online ahead of print. doi: 10.1002/advs.202522929 (PMC13325833; doi:10.1002/advs.202522929)
Supplement: Supplementary file 1 — Supporting File: advs74883‐sup‐0001‐SuppMat.docx. [file ADVS-9999-e22929-s001.docx]

**Title:** GHRHR Deficiency Enhances Retinal Ganglion Cell Survival and Visual Functions in Experimental Glaucoma by Inhibiting Ferroptosis

*Yan Tong^1,2,7^, Ming Ho Yam^3^, Jiaxin Zhang^1^, Lin Du^1^, Linbin Zhou^1^, Yolanda Wong Ying Yip^1^, Bo Man Ho^1^, Hemlata Bisnauthsing^1^, Jiahui Li^1^, Ivan Kong^1^, Shushu Xu^1^, Fu Changzhen^1,2^, Karl KH So^3^, Joaquim SL Vong^3^, Ling-Ping Cen^2,4,5,6^,* *Ming-Ming Yang^7^, Khazeema Yousaf^8,9^, Mai Har Sham^3^, Sun On Chan^3^, Poemen P. Chan^1,2,10^, Chi Pui Pang^1,2,10^, Clement C. Tham^1,2,10^, Jing Na He^1*^, Jian Li^1,11*^, Wai Kit Chu^1,2,10*^.*

**
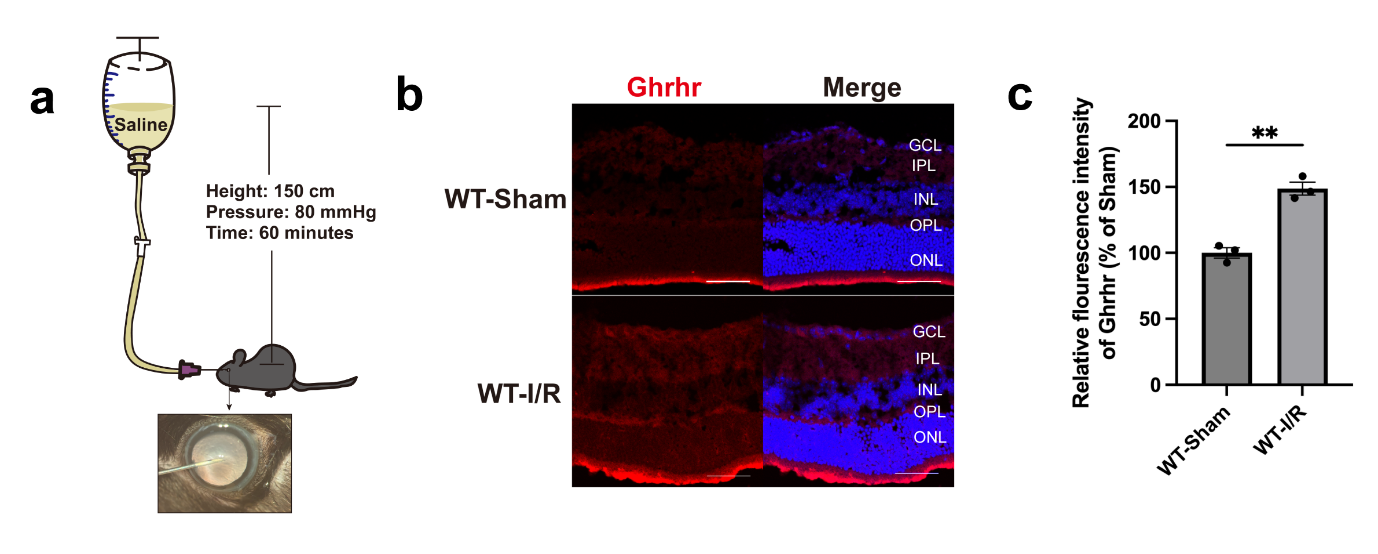
**

**Supplementary Figure 1. Elevated expression of GHRHR in the mouse retinas after I/R injury**. **a**. Schematic representation of the retinal I/R model. **b.** Representative retinal immunofluorescence staining of Ghrhr (red), counterstained with DAPI (blue). Scale bars represent 50 μm. **c**. Relative fluorescence intensity of Ghrhr protein expression in retinal sections (n=3 in each group). All results are presented as the mean ± SEM; *P*-values are calculated using unpaired two-tailed Student’s t-tests; significance levels are denoted as **P <0.01.


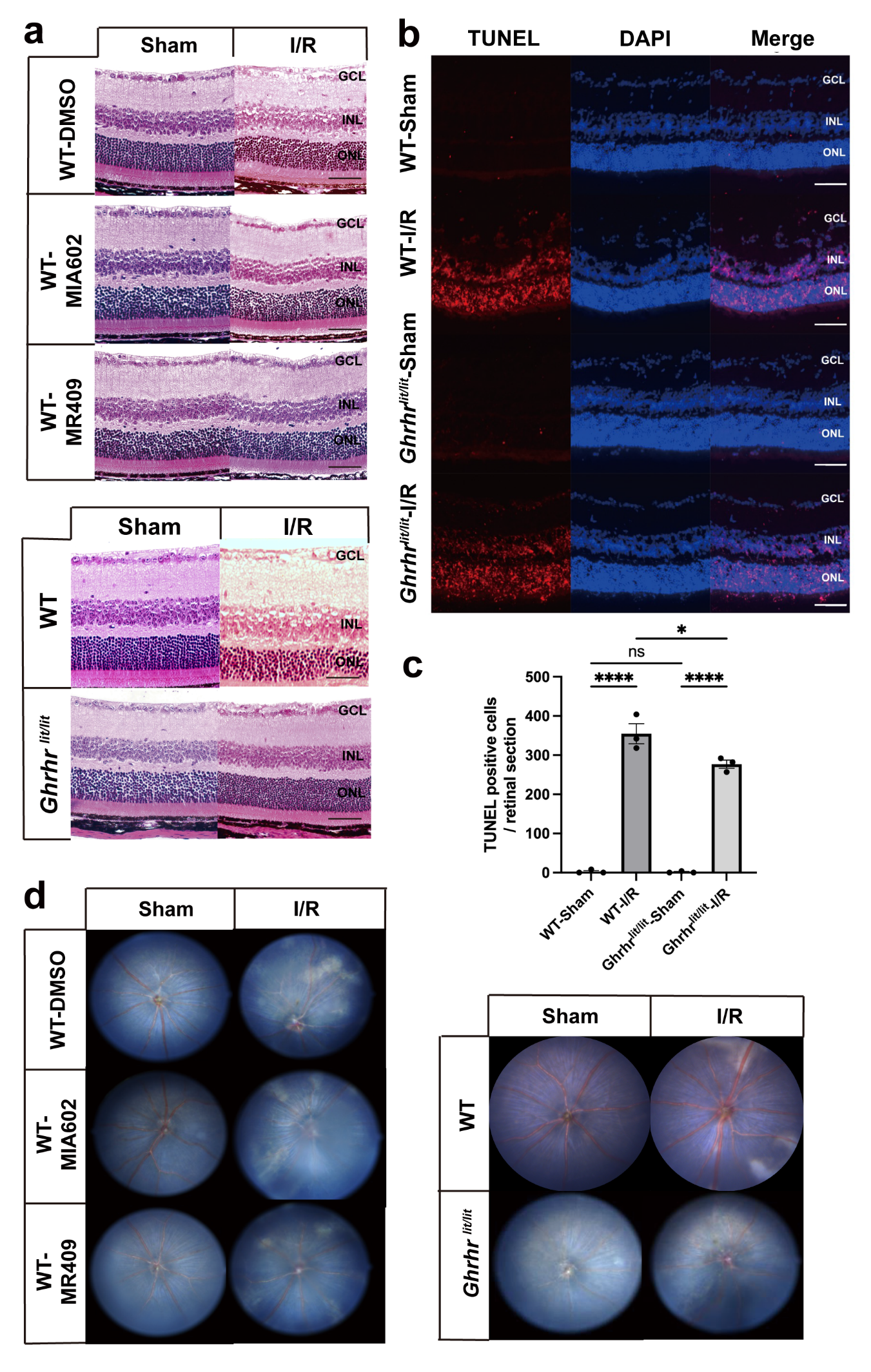


**Supplementary Figure 2. GHRHR deficiency protected retinal morphology of mice from I/R injury**. **a**. Representative H&E staining from mice treated with DMSO, MIA602 or MR409, and *Ghrhr^lit/lit^* mice with or without I/R. **b-c**. Representative TUNEL staining images and quantification of retinal sections (n=3 in each group). **d**. Representative fundus images from control mice, mice treated with MIA602 and MR409, and *Ghrhr^lit/lit^* mice with or without I/R. All the results are presented as the mean ± SEM; *P*-values are calculated using one-way ANOVA with Tukey’s correction; significance levels are denoted as *P <0.05 and ****P <0.0001. ns represents no significance. Scale bars, 50 μm in (a and b).


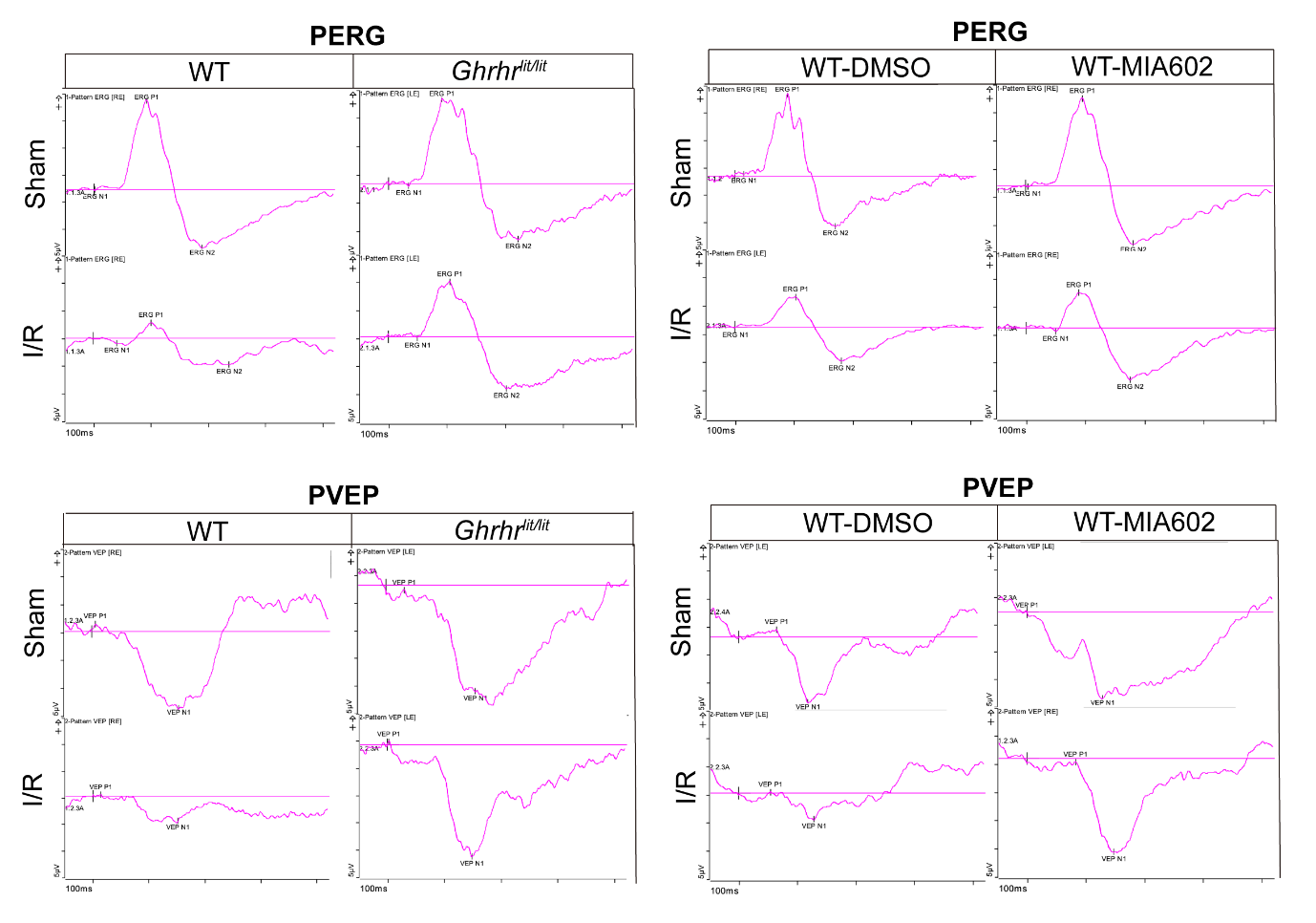


**Supplementary Figure 3. Representative waveforms of PERG and PVEP in different groups.**

**Table S1**. Cell numbers and percentages of distinct cell clusters in retinas of WT-I/R and *Ghrhr^lit/lit^*-I/R groups

| **Cell types** | **Cell numbers** | | **Percentages (%)** | |
| --- | --- | --- | --- | --- |
|  | WT-I/R  (11,787 cells) | *Ghrhr^lit/lit^*-I/R  (9,817 cells) | WT-I/R | *Ghrhr^lit/lit^* -I/R |
| Rod | 2,343 | 2,002 | 19.9 | 20.4 |
| Rod bipolar | 931 | 706 | 7.9 | 7.2 |
| Cone | 992 | 798 | 8.4 | 8.1 |
| Cone bipolar (off) | 1,175 | 960 | 10.0 | 9.8 |
| Cone bipolar (on) | 1,593 | 1,379 | 13.5 | 14.0 |
| Glycinergic amacrine cell 1 | 1,024 | 958 | 8.7 | 9.8 |
| Glycinergic amacrine cell 2 | 292 | 229 | 2.5 | 2.3 |
| GABAergic amacrine cell | 1,530 | 1,291 | 13.0 | 13.2 |
| Astrocytes | 31 | 27 | 0.3 | 0.3 |
| Macroglia | 1,510 | 1,173 | 12.8 | 11.9 |
| Horizontal cell | 44 | 31 | 0.4 | 0.3 |
| Retinal ganglion cell | 74 | 75 | 0.6 | 0.8 |
| Retinal pigment epithelium | 78 | 69 | 0.7 | 0.7 |
| Endothelial cell | 96 | 90 | 0.8 | 0.9 |
| Immune cells | 74 | 29 | 0.6 | 0.3 |

**
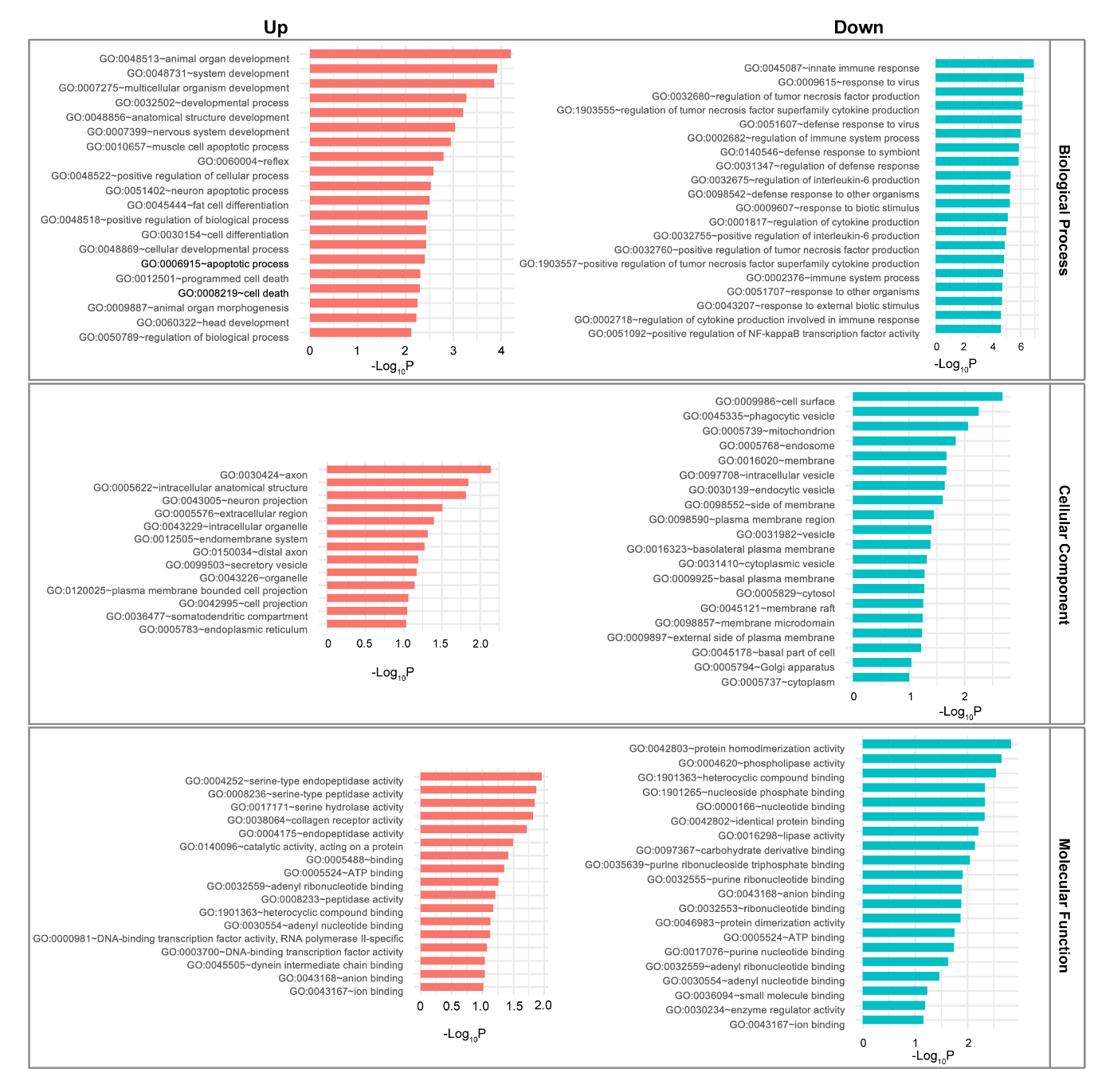
**

**Supplementary Figure 4. GO analysis of upregulated and downregulated genes in *Ghrhr^lit/lit^* -I/R retinas compared to WT-I/R retinas.** The levels of gene expression (reads per kilobase per million reads) were depicted on a log_10_ scale.


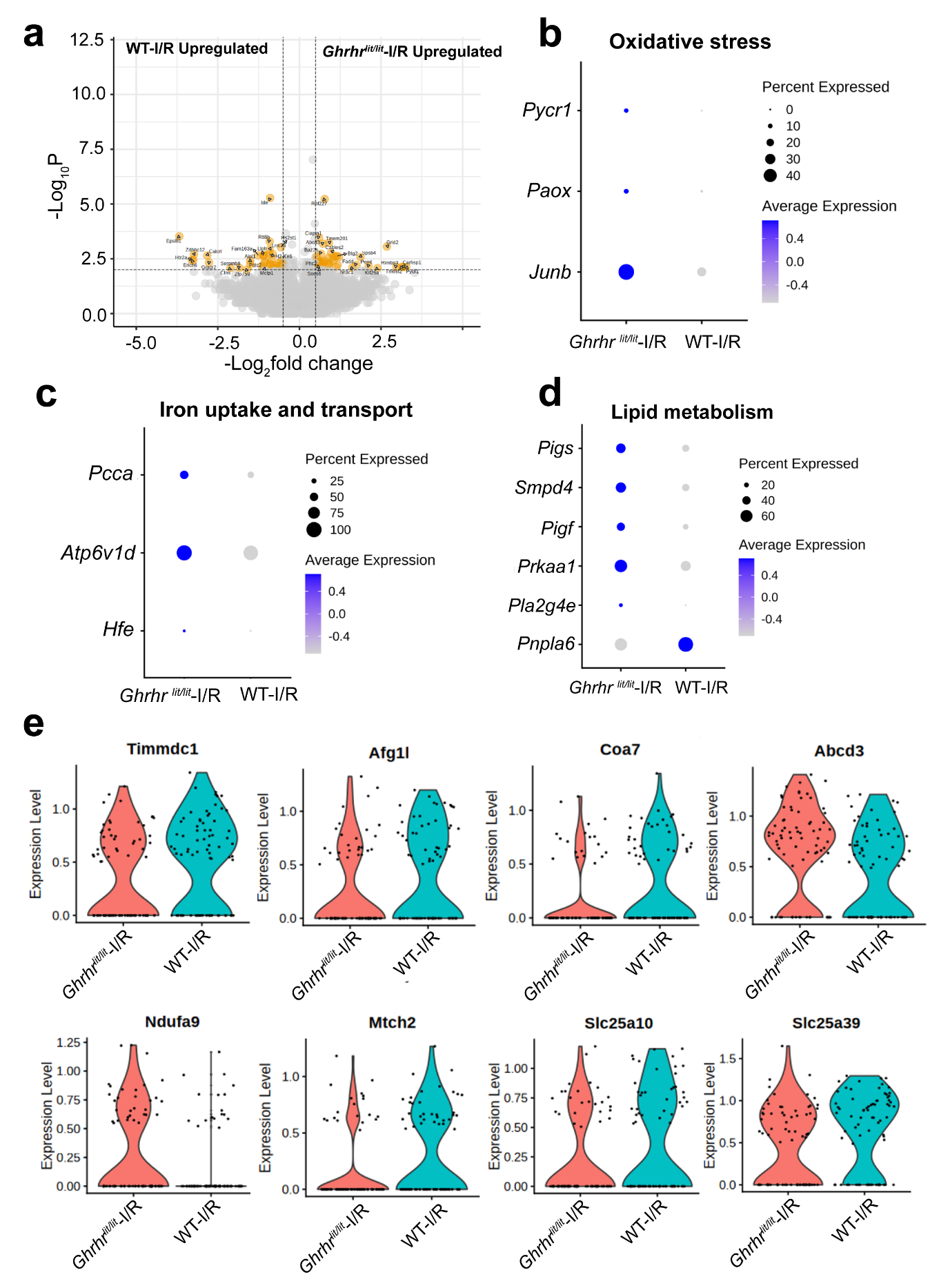


**Supplementary Figure 5. Single-cell RNA-seq identifies DEGs in RGCs of *Ghrhr^lit/lit^* mice following I/R. a.** Volcano plot illustrating DEGs in the RGC subcluster from *Ghrhr^lit/lit^*-I/R and WT-I/R retinas. **b-d.** Feature plots depicting DEGs related to oxidative stress (**b**), iron uptake and transport (**c**), and lipid metabolism (**d**) within RGCs. **e**. Violin plots comparing mitochondria function-related DEGs in RGCs between *Ghrhr^lit/lit^* -I/R and WT-I/R groups.

**
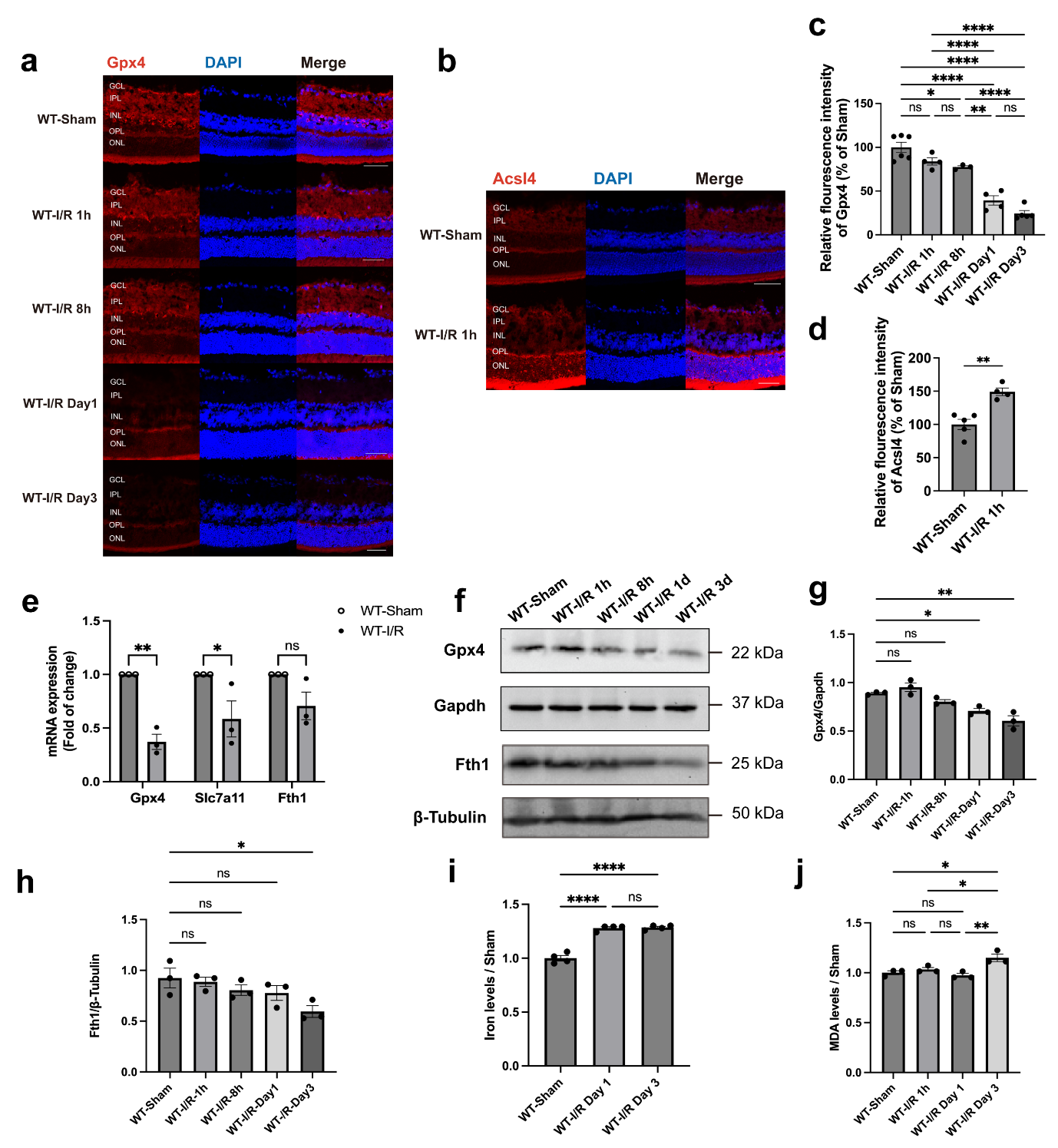
**

**Supplementary Figure 6. I/R injury induces ferroptosis in mouse retinas**. **a-b**. Representative immunofluorescence staining of *Gpx4* and *Acsl4* (red) in retinal sections, counterstained with DAPI. **c-d**. Relative florescence intensity of *Gpx4* and *Acsl4* protein expression in retinal section (n=3 in each group). **e**. Relative mRNA expression levels of *Gpx4*, *Slc7a11*, and *Fth1*, quantified and normalized to *Gapdh*. Fold change is relative to WT-Sham mice (n=3). **f-h**. Western blot analysis of Gpx4 and Fth1 in retinas from sham and I/R treated mice (n=3). **i-j**. Measurement of tissue iron (i) and MDA (j) in sham and I/R treated retinas (n=3). All results are presented as the mean ± SEM; *P*-values are calculated using one-way ANOVA with Tukey’s correction (**c**, **g, h, i, j**) or unpaired two-tailed Student’s t-tests (**d**, **e**); significance levels are denoted as *P <0.05, **P <0.01, ***P <0.001 and ****P <0.0001. ns represents no significance. Scale bars, 50 μm in (a) and (b).

**
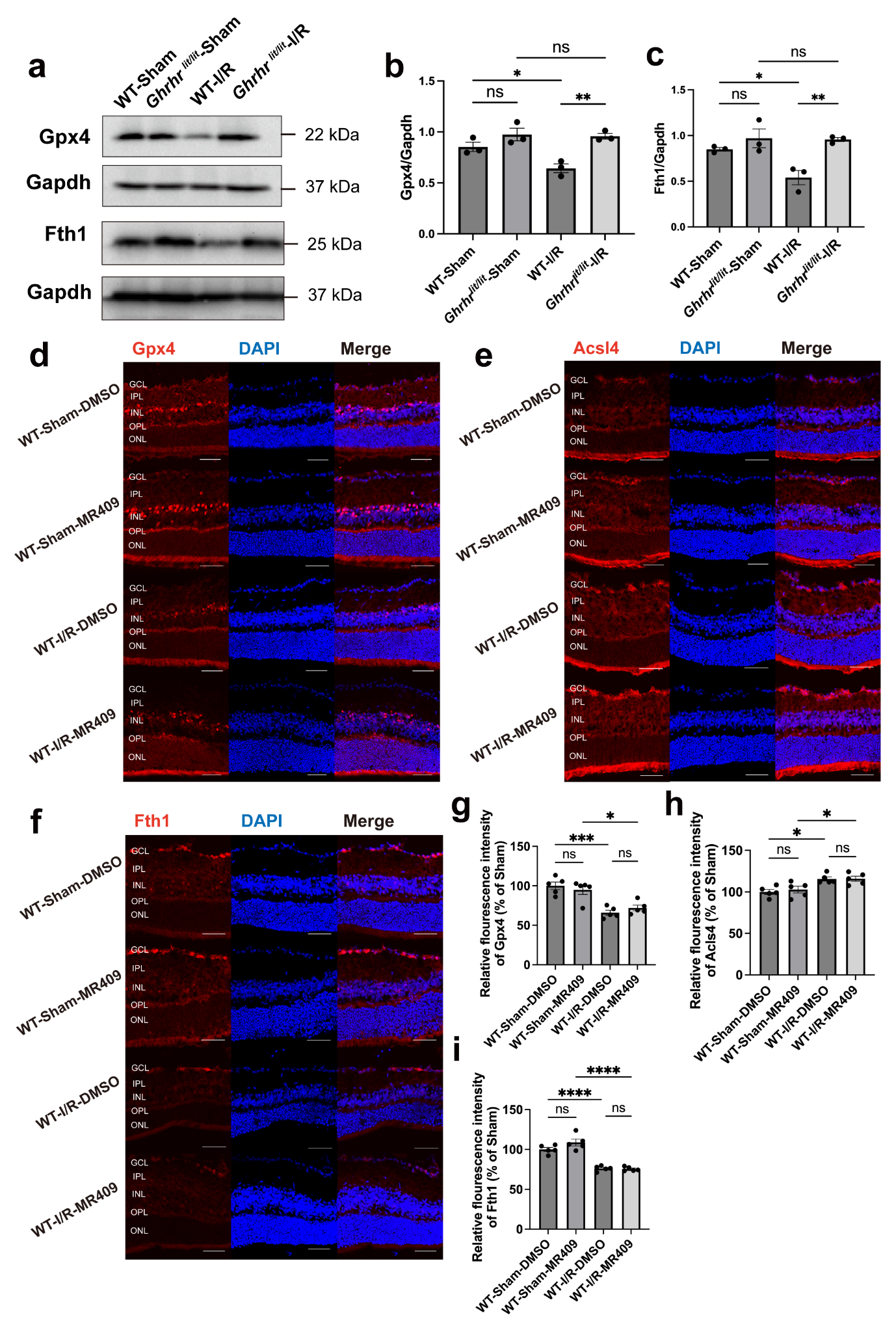
**

**Supplementary Figure 7. GHRHR deficiency mitigates I/R-induced RGC injury by suppressing ferroptosis. a-c.** Western blot and densitometry analysis of Gpx4 and Fth1 expression in retinas in WT and *Ghrhr^lit/lit^* groups three days post I/R. N=3 per group. **d-f**. Representative retinal immunofluorescence staining for Gpx4, Acsl4, and Fth1 (red), counterstained with DAPI. **g-i**. Relative florescence intensities of Gpx4, Acsl4, and Fth1 protein expression in retinal sections. N=5 per group. *P*-values are calculated using one-way ANOVA with Tukey’s correction. Significance levels are denoted as *P <0.05, **P <0.01. ns represents no significance. Scale bars, 50 μm in (**d-f**).

**
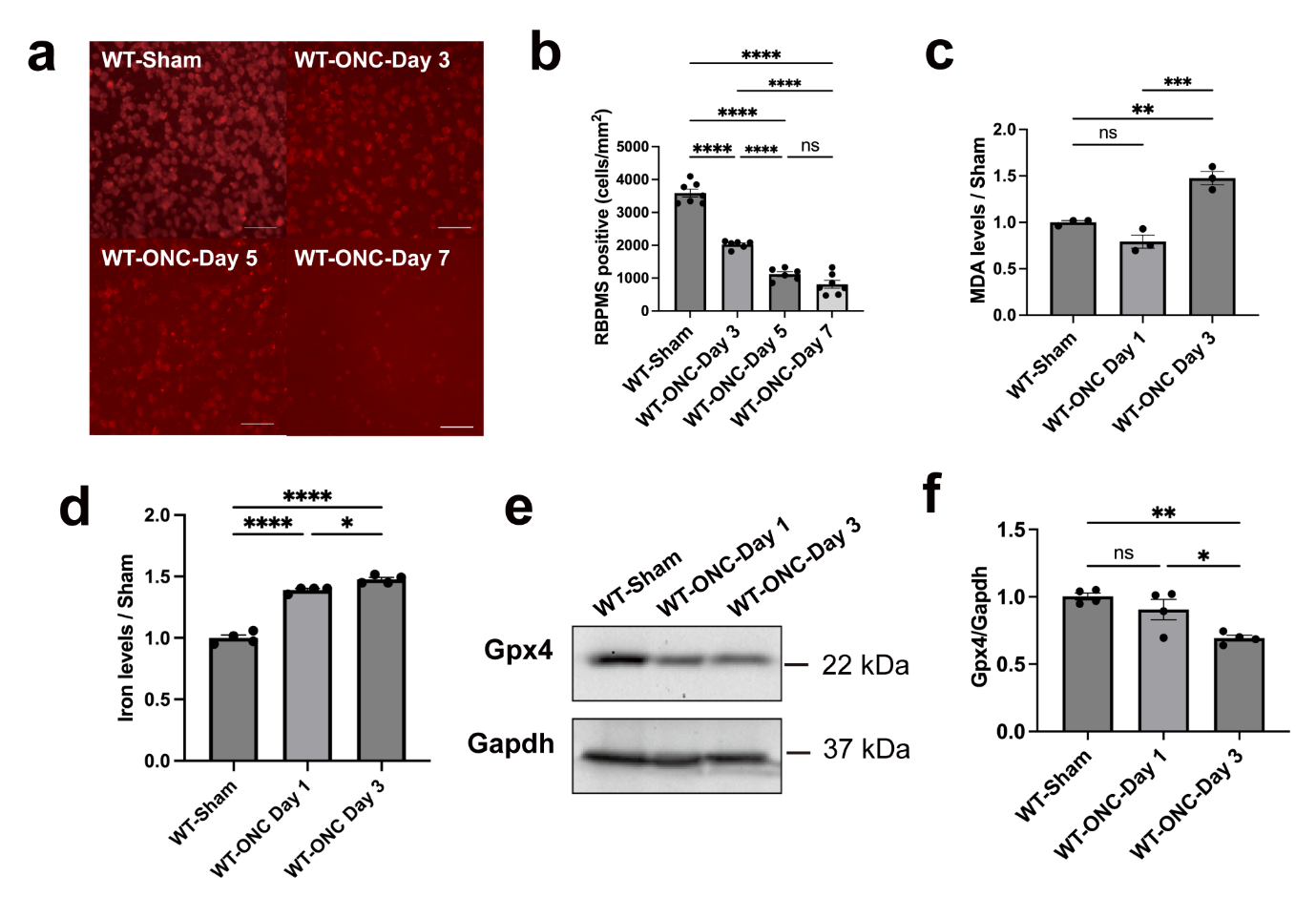
**

**Supplementary Figure 8. Ferroptosis induction in mouse retinas after ONC injury. a-b**. Immunofluorescent staining (**a**) and quantification (**b**) of RBPMS-positive cells in sham and ONC-challenged retinas at 3-, 5-, and 7-day post-injury. N=6-7 per group. **c-d.** Measurement of MDA (**c**) and tissue iron levels (**d**) in sham and ONC-challenged retinas. N=3-4 per group. **e-f**. Western blot and densitometry analysis of Gpx4 expression in sham and ONC-challenged retinas. N=4 per group. *P*-values are calculated using one-way ANOVA with Tukey’s correction. Significance levels are denoted as *P <0.05, **P <0.01 and ****P <0.0001. ns represents no significance. Scale bars, 50 μm in (a).


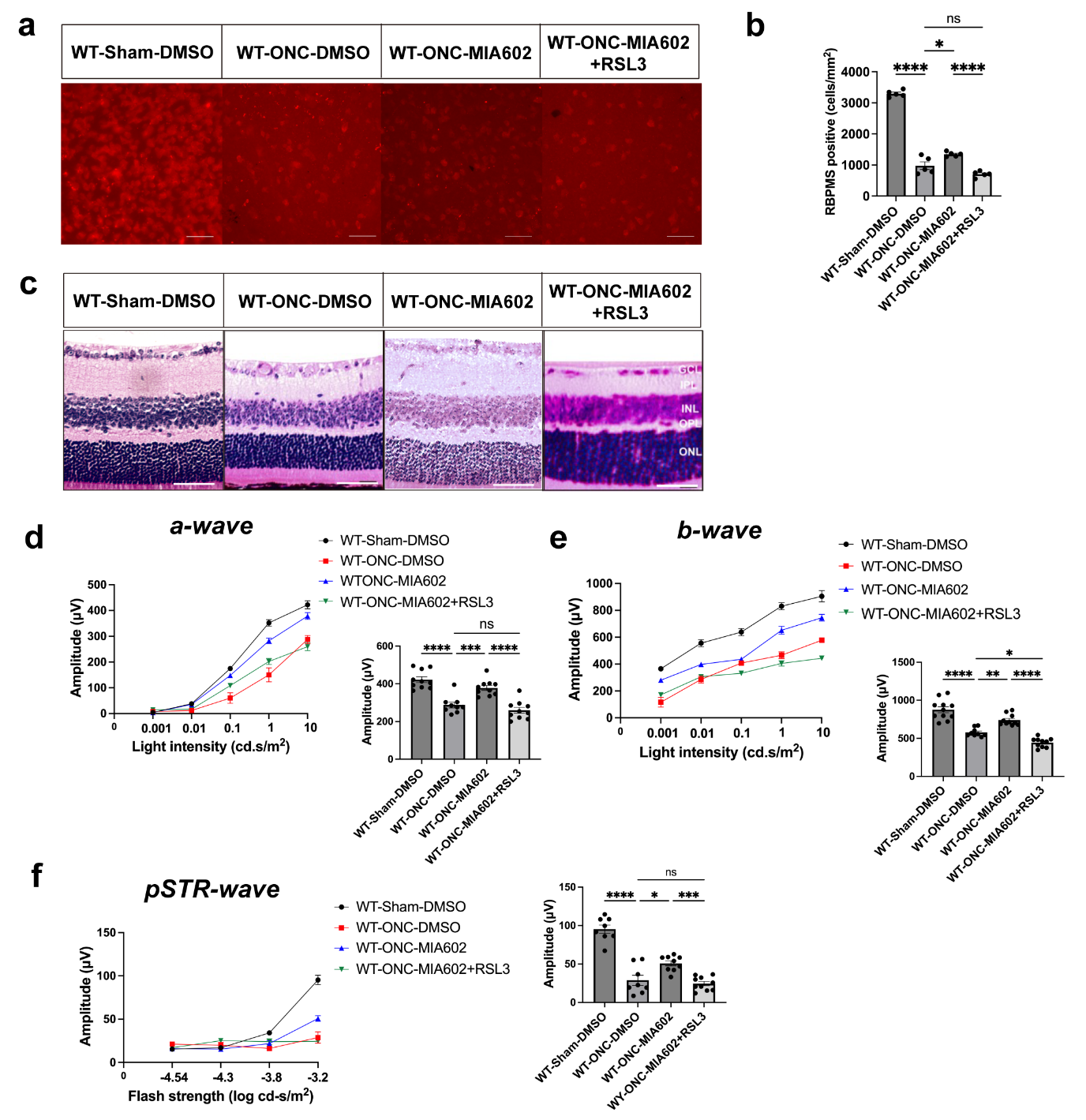


**Supplementary Figure 9. The co-treatment of ferroptosis inducer RSL3 abolished the protective effects of GHRHR inhibition after ONC injury**. **a-b**. Representative images (a) and quantification (b) of RBPMS-positive cells in retinal whole-mounts from GHRHR inhibited eyes plus RSL3 co-treatment (N=5 per group) **c**. Representative H&E staining of ONC eyes co-treated with MIA602 and RSL3. **d-f**. Amplitudes of scotopic full-field ERG a-waves (**d**), b-waves (**e**), and pSTR (**f**) recorded 5 days post-ONC challenge (N=6-11 per group). *P*-values are calculated using one-way ANOVA with Tukey’s correction. Significance levels are denoted as *P <0.05, **P <0.01, ***P <0.001 and ****P <0.0001. ns represents no significance. Scale bars, 50 μm in (a and c).

**Table S2**. Primary antibodies used for immunostaining (IF) and western blotting (WB)

| **Antibody name** | **Company** | **Cat. No** | **Working dilution** |
| --- | --- | --- | --- |
| Gpx4 | Abcam | ab125066 | 1:200 (IF); 1:1000 (WB) |
| Acsl4 | Abcam | Ab155282 | 1:200 (IF) |
| Fth1 | Cell Signaling Technology | 4393 | 1:1000 (WB) |
| Slc7a11 | Abcam | Ab175186 | 1:200 (IF) |
| Rbpms | Gene Tex | GTX118619 | 1:500 (IF) |
| Alexa Fluor® 594-conjuagated Thy1 | Santa Cruz | sc-53456 AF594 | 1:200 (IF) |
| CoraLite®488-conjugated Rbpms | Proteintech | CL488-15187 | 1:200 (IF) |

**Table S3.** List of primer pairs used in qRT-PCR

| **Genes** | **Forward primer (5'-3')** | **Reverse primer (5'-3')** |
| --- | --- | --- |
| *Gpx4* | ATAAGAACGGCTGCGTGGTGAAG | TAGAGATAGCACGGCAGGTCCTTC |
| *Slc7a11* | CTATTTTACCACCATCAGTGCG | ATCGGGACTGCTAATGAGAATT |
| *Fth1* | TAAAGAAACCAGACCGTGATGA | ATTCACACTCTTTTCCAAGTGC |
| *β-actin* | CTACCTCATGAAGATCCTGACC | CACAGCTTCTCTTTGATGTCAC |
